# Spatial expression patterns of genes encoding sugar sensors in leaves of C_4_ and C_3_ grasses

**DOI:** 10.1093/aob/mcad057

**Published:** 2023-04-27

**Authors:** Urs F Benning, Lily Chen, Alexander Watson-Lazowski, Clemence Henry, Robert T Furbank, Oula Ghannoum

**Affiliations:** Hawkesbury Institute for the Environment, Western Sydney University, Hawkesbury Campus, New South Wales 2753, Australia; Hawkesbury Institute for the Environment, Western Sydney University, Hawkesbury Campus, New South Wales 2753, Australia; Harper Adams University, Edgmond, TF10 8NB, UK; Hawkesbury Institute for the Environment, Western Sydney University, Hawkesbury Campus, New South Wales 2753, Australia; ARC Centre of Excellence for Translational Photosynthesis, Research School of Biology, Australian National University, Canberra, Australian Capital Territory 2601, Australia; Hawkesbury Institute for the Environment, Western Sydney University, Hawkesbury Campus, New South Wales 2753, Australia

**Keywords:** C_3_ and C_4_ photosynthesis, hexokinase (HXK), grasses, sink, source, sugar sensing genes, SNF1-related kinase 1 (SnRK1), target of rapamycin (TOR), trehalose-6-phosphate (T6P)

## Abstract

**Background and Aims:**

The mechanisms of sugar sensing in grasses remain elusive, especially those using C_4_ photosynthesis even though a large proportion of the world's agricultural crops utilize this pathway. We addressed this gap by comparing the expression of genes encoding components of sugar sensors in C_3_ and C_4_ grasses, with a focus on source tissues of C_4_ grasses. Given C_4_ plants evolved into a two-cell carbon fixation system, it was hypothesized this may have also changed how sugars were sensed.

**Methods:**

For six C_3_ and eight C_4_ grasses, putative sugar sensor genes were identified for target of rapamycin (TOR), SNF1-related kinase 1 (SnRK1), hexokinase (HXK) and those involved in the metabolism of the sugar sensing metabolite trehalose-6-phosphate (T6P) using publicly available RNA deep sequencing data. For several of these grasses, expression was compared in three ways: source (leaf) versus sink (seed), along the gradient of the leaf, and bundle sheath versus mesophyll cells.

**Key Results:**

No positive selection of codons associated with the evolution of C_4_ photosynthesis was identified in sugar sensor proteins here. Expressions of genes encoding sugar sensors were relatively ubiquitous between source and sink tissues as well as along the leaf gradient of both C_4_ and C_3_ grasses. Across C_4_ grasses, *SnRK1β1* and *TPS1* were preferentially expressed in the mesophyll and bundle sheath cells, respectively. Species-specific differences of gene expression between the two cell types were also apparent.

**Conclusions:**

This comprehensive transcriptomic study provides an initial foundation for elucidating sugar-sensing genes within major C_4_ and C_3_ crops. This study provides some evidence that C_4_ and C_3_ grasses do not differ in how sugars are sensed. While sugar sensor gene expression has a degree of stability along the leaf, there are some contrasts between the mesophyll and bundle sheath cells.

## INTRODUCTION

Given that C_4_ species fix carbon and synthesize carbohydrates using a two-cell system compared with C_3_ species, which use a single cell, it remains unclear if the sugars produced are sensed differently between them. C_4_ photosynthesis evolved ~35 million years ago in response to a period of low atmospheric CO_2_, evolving in 62 independent lineages (Sage, 2004, 2017; Sage *et al.*, 2011). Many agronomically important cereals, such as *Zea mays* (maize), *Sorghum bicolor* (sorghum), *Panicum virgatum* (switchgrass) and millets (such as *Setaria italica*), utilize C_4_ photosynthesis. This evolution has led to major changes in gene expression, leaf morphology, biochemistry and the compartmentalization of photosynthetic reactions (Dengler and Nelson, 1999; Von Caemmerer and Furbank, 2003; McKown and Dengler, 2007; Muhaidat *et al.*, 2007; Emms *et al.*, 2016; Furbank and Kelly, 2021).

This compartmentalization of C_4_ photosynthesis is enabled by a specialized leaf anatomy, known as Kranz anatomy, whereby mesophyll cells are arranged in a concentric layer around the bundle sheath cells (Haberlandt, 1904; Hattersley, 1984). In the mesophyll of C_4_ leaves, CO_2_ is hydrated into bicarbonate and is initially fixed by phospho*enol*pyruvate (PEP) carboxylase (PEPC), using PEP as a CO_2_ acceptor (Hatch and Slack, 1966; Furbank and Hatch, 1987). Oxaloacetate (OAA) is then produced and rapidly converted to two possible C_4_ acids, malate or aspartate. These acids diffuse to the bundle sheath via the abundant plasmodesmatal connections, where they are decarboxylated, releasing CO_2_ to be refixed by ribulose-1,5-bisphosphate carboxylase-oxygenase (Rubisco) (Danila *et al.*, 2016, 2018). The compartmentalization of the photosynthetic enzymes, the high PEPC/Rubisco activity ratio and the low permeability of the bundle sheath cell wall elevate CO_2_ concentration around Rubisco, leading to near CO_2_ saturation and reduced photorespiration (Hatch, 1987; Ghannoum *et al.*, 2000; Von Caemmerer and Furbank, 2003; Danila *et al.*, 2021).

During the evolution of C_4_ photosynthesis, the expression of numerous genes was adjusted to enable distinct spatial separation, or altered regulation, relative to expression patterns seen in species that utilize C_3_ photosynthesis (Hibberd and Covshoff, 2010; Westhoff and Gowik, 2010; Christin and Osborne, 2014). Some examples include the targeted expression of PEPC and the confinement of carbonic anhydrase to the mesophyll cell in C_4_ plants as well as differences in Rubisco catalytic efficiencies between the two photosynthetic types (Gowik *et al.*, 2004; Tetu *et al.*, 2007; Tanz *et al.*, 2009; Whitney *et al.*, 2011; Ludwig, 2016). The evolution of photosynthesis into a two-cell process in C_4_ plants has also resulted in the spatial partitioning of carbohydrate production. One of the main products of photosynthesis, triose phosphate, is used in the synthesis of soluble sugars such as glucose and sucrose, substrates that can then be synthesized into the storage carbohydrate starch. Generally, in C_4_ plants sucrose biosynthesis occurs in the mesophyll, while starch synthesis occurs predominantly in bundle sheath chloroplasts (Lunn and Furbank, 1997, 1999; Lunn, 2007; Furbank and Kelly, 2021). In leaves of C_3_ species these processes occur almost exclusively in the mesophyll. Carbohydrates are moved from the photosynthetic source leaves to the heterotrophic sink tissues such as seeds, stems, roots and young leaves for growth and development.

Photosynthesis and sink demand are tightly coordinated through metabolic feedback and signalling mechanisms (Blechschmidt-Schneider *et al.*, 1989; Sheen, 1990). Sugar signalling integrates sugar production with plant development and environmental cues (Rolland *et al.*, 2006). To date, there is a limited understanding of the molecular mechanisms underlying these feedback regulations in C_4_ plants. C_4_ species evolved in arid and warmer climates, conditions that may have also imposed specific selective pressures on aspects of sugar sensing. There is also evidence showing that the high photosynthetic activity in C_4_ leaves can lead to the accumulation of higher levels of sugars, relative to C_3_ species (Henry *et al.*, 2020). Carbohydrate synthesis, metabolism and export differ in several ways between C_4_ and C_3_ photosynthetic species. As mentioned, sucrose and starch synthesis is compartmentalized in leaves of C_4_ grasses (Lunn and Furbank, 1999). In addition, large metabolite pools are required to maintain a high concentration gradient across the mesophyll–bundle sheath interface with higher plasmodesmatal connections in C_4_ grasses, allowing fast metabolite exchange and efficient carbon concentration around Rubisco (Leegood, 2002; Danila *et al.*, 2016). Part of the 3-phosphoglycerate (PGA) produced by the Calvin cycle in bundle sheath cells is reduced in mesophyll cells due to lower photosystem II activity in the bundle sheath. Furthermore, there has been recent evidence that suggests that C_4_ grasses have evolved sugar transporters, using a different strategy compared with C_3_ grasses (Emms *et al.*, 2016; Bezrutczyk *et al.*, 2018; Chen *et al.*, 2022; Hua *et al.*, 2022). These factors suggest that sugar sensing may differ between C_4_ and C_3_ plants, and between the mesophyll and bundle sheath cells.

Three putative sugar sensor kinase proteins are known: target of rapamycin (TOR), SNF1-related kinase 1 (SnRK1), hexokinase (HXK) and the sugar-sensing metabolite trehalose-6-phosphate (T6P). TOR functions as a protein kinase and is a part of the TOR complex (TORC), which also includes RAPTOR (regulatory-associated protein of TOR 1) and LST8 (lethal with sec thirteen 8). These additional proteins can act as regulatory components of the TORC (Xiong and Sheen, 2014). In *Arabidopsis thaliana*, it has been established that the glucose–TOR signalling network can regulate numerous essential processes (Xiong and Sheen, 2012; Xiong *et al.*, 2013). Hexokinase was one of the first proteins for which a direct link between sugar sensing and photosynthesis was established (Moore *et al.*, 2003). Several homologues, such as AtHXK1 in *Arabidopsis* and OsHXK5 and OsHXK6 in rice (*Oryza sativa*), have been established as sugar sensor proteins (Moore *et al.*, 2003; Cho *et al.*, 2009*a*). There has also been evidence for SnRK1 as a sugar-sensing protein in plants (Jossier *et al.*, 2009). The SnRK1 complex (SnRK1C) is made up of four subunits – the catalytic subunit (α), two regulatory subunits (β,γ) and a hybrid plant-specific subunit (βγ) – and can be involved in plant–pathogen interactions (Bouly *et al.*, 1999; Lumbreras *et al.*, 2001; Gissot *et al.*, 2005, 2006). SnRK1 is thought to be upregulated when conditions are unfavourable for the plant (Baena-González *et al.*, 2007; Zhang *et al.*, 2009). There has been some evidence that SnRK1 is involved in the regulation of photosynthesis genes as the overexpression of *KIN10* (the gene encoding the SnRK catalytic subunit in *Arabidopsis*) causes a downregulation of photosynthetic genes. Furthermore, SnRK1 is inhibited by T6P (the precursor to the disaccharide trehalose), but not by other sugars. In plants, T6P can only be made when sufficient levels of sucrose are present (Lunn *et al.*, 2006), and therefore acts as a signalling molecule for sucrose and correlates with active growth (Schluepmann *et al.*, 2003; Martínez-Barajas *et al.*, 2011; Lunn et al., 2006, 2014). Trehalose phosphate synthase (TPS) is responsible for the synthesis of T6P, and T6P can subsequently be converted to trehalose via trehalose phosphate phosphatase (TPP) (Ponnu *et al.*, 2011; Paul *et al.*, 2020). Trehalose can then be broken back down into its glucose units by trehalase (TRE). While trehalose is found at relatively low levels in plants, it is thought that trehalose metabolism plays an important regulatory role (Goddijn and Smeekens, 1998).

Due to innate differences and the complexity of the signalling network, it is plausible to hypothesize that photosynthetic types may sense sugars differently. Sugar sensors may have evolved to accommodate the two-cell compartmentalization of C_4_ photosynthesis. In this study, publicly available transcriptome data from C_3_ and C_4_ grasses were used to investigate the expression of putative genes encoding components of each sugar sensor. The overall aim was to determine if there were differences in expression patterns between C_3_ and C_4_ grasses that might alter how sugar is perceived. Data were used to (1) determine if there were C_4_-specific residues in the sugar-sensing genes associated with the evolution of C_4_ photosynthesis, and (2) compare the transcript abundance (a) between the leaf (source) and seed (sink) in C_4_ and C_3_ grasses, (b) along the leaf gradient of C_4_ and C_3_ grasses, where a single leaf undergoes a sink (base)–source (tip) transition during development (Jones and Eagles, 1962; Turgeon and Webb, 1976; Harn *et al.*, 1993; Kölling *et al.*, 2013; Wang *et al.*, 2014; Chen *et al.*, 2022), and (c) between the bundle sheath and mesophyll cells of C_4_ grasses, to investigate whether there was preferential expression to one photosynthetic cell type. This approach can shed light on those sugar sensors that might be linked with photosynthesis (source tissue).

## MATERIALS AND METHODS

### C_4_ and C_3_ grass species utilized

Sequences, transcript expression data or both were extracted from eight C_4_ grasses (*Panicum hallii*, *Panicum miliaceum*, *Panicum antidotale*, *Sorghum bicolor*, *Setaria italica*, *Setaria viridis*, *Saccharum spontaneum* and *Zea mays*) and six C_3_ grasses (*Steinchisma laxum*, *Hymenachne amplexicaulis*, *Cyrtococcum patens*, *Panicum bisulcatum*, *Brachypodium distachyon* and *Oryza sativa*). The species, transcriptomes and raw RNA sequencing data that were used for each aspect of this study are summarized in Supplementary Data Table S1.

### Obtaining and mining publicly available assemblies

Where genomes were publicly available, the associated annotations and transcriptomes were obtained and mined for genes of interest. Data were downloaded from Phytozome v13 (Goodstein *et al.*, 2012) or https://www.ncbi.nlm.nih.gov/ for the following assemblies: *Z. mays* v4 (Jiao *et al.*, 2017), *S. spontaneum* (Zhang *et al.*, 2018), *S. bicolor* v3.1.1 (McCormick *et al.*, 2018), *S. italica* v2.2 (Bennetzen *et al.*, 2012; Zhang *et al.*, 2012), *S. viridis* v2.1 (Bennetzen *et al.*, 2012), *P. hallii* v3.2 (Lovell *et al.*, 2018), *O. sativa* v7 (Ouyang *et al.*, 2007) and *B. distachyon* v2.1 (Vogel *et al.*, 2010). Known protein sequences from *Arabidopsis*, *Z. mays* and/or *O. sativa* were used to identify homologues in the other species through the Phytozome v13 online interface (http://phytozome.jgi.doe.gov; Goodstein *et al.*, 2012). The gene IDs for each of the sequences from each species can be found in Supplementary Data File S1.

### De novo *assembly of RNA-sequencing reads*

A *de novo* transcriptome assembly was built for those species that had no publicly available genomes at the time of analysis using published RNA-sequencing (RNA-Seq) data. All RNA-Seq data sets were obtained from https://www.ncbi.nlm.nih.gov/ or https://www.ebi.ac.uk/ using the project’s associated accession number (Supplementary Data Table S1). Adapter sequences were first removed from all RNA-Seq reads using Trimmomatic (Bolger *et al.*, 2014). The Trinity default pipeline was then implemented to create the *de novo* assemblies, each consisting of a set of contiguous sequences (contigs) for each species (Grabherr *et al.*, 2011; Haas *et al.*, 2013). For each *de novo* assembly, annotation of contigs was required to identify genes of interest. For this, *de novo* assemblies were loaded into Geneious Prime, 2022.2 (https://www.geneious.com; Kearse *et al.*, 2012), and nucleotide databases were created using the inbuilt NCBI BLAST tool. The NCBI tool within Geneious Prime 2022.2 was then used to carry out BLASTN queries, using sequences of genes of interest from *S. viridis* (Bennetzen *et al.*, 2012) and *O. sativa* (Ouyang *et al.*, 2007) transcriptomes, for identification.

### Estimation of transcript abundance

RNA-Seq reads obtained (Supplementary Data Table S1) were quantified using the quasi-align mode in Salmon (Patro *et al.*, 2017). A mapping-based index was created for each transcriptome or *de novo* transcriptome. Trimmed reads were then mapped to the relevant index using the default settings of the quant command in mapping-based mode within Salmon. This produced normalized transcripts per million (TPM) values for each transcript or contig (Kearse *et al.*, 2012).

### Protein sequence alignment and phylogeny

Protein alignments and phylogenetic trees were created to visualize homology using Geneious Prime 2022.2 (https://www.geneious.com; Kearse *et al.*, 2012). Alignments were created using Multiple Alignment with Fast Fourier Transform (MAFFT) using a G-INS-i algorithm and BLOSUM62 matrix (with a 1.53 gap penalty and 0.123 offset value) for the scoring (Katoh and Standley, 2013). Phylogenetic trees were built using Randomized Axelerated Maximum Likelihood (RaxML) using 1000 bootstrap replicates (Stamatakis, 2006, 2014).

### Positive selection analysis using CodeML

To investigate for evidence of positive selection in genes of interest, CodeML was implemented to test residues for selection (Zhang *et al.*, 2005). For this analysis, species were selected to ensure C_4_ lineages were dispersed phylogenetically between C_3_ species (Supplementary Data Table S1). Phylogenetic trees created using RaxML were processed as Newick files and annotated (using #1 to denote a foreground branch) to test the hypothesis of selection in foreground branches. Those branches labelled as foreground were those that contain a C_4_ species, hence the following analyses test whether there is any positive selection associated with the evolution of C_4_ photosynthesis (C_4_-specific selection). CodeML was then used to test the ratio of non-synonymous to synonymous substitutions (dN/dS ratio; ω) under two scenarios: (1) a null model where all codons evolve under either purifying selection (ω < 1) or relaxed selection (ω = 1); and (2) sites evolve under purifying or neutral selection in the whole tree, except for foreground branches, where they evolve under positive selection (ω > 1). These scenarios were compared to estimate the posterior probability of each base evolving under positive selection using a Bayes empirical model. These scenarios were tested in pamlX, a package housing CodeML (Xu and Yang, 2013).

### Source-to-sink expression data

Publicly available transcript expression data from the leaf and seed were downloaded for two C_4_ grasses and two C_3_ grasses (Supplementary Data Table S1). Except for data associated with *P. miliaceum*, all data were microarray data, where expression was represented as robust multichip average (RMA)-normalized expression values. For *P. miliaceum*, the dataset was RNA-Seq, and therefore TPM values were extracted as described above. Using these data, the leaf expression values were divided by the seed expression values to obtain a source-to-sink ratio. There were three biological replicates used for each study. Since there were only two species surveyed for each photosynthetic type, the biological replicates were used as the *n* value when representing the source-to-sink ratio for C_4_ and C_3_ grasses. Therefore *n* = 6 for the ratios in this experimental study. These values can be found in Supplementary Data File S2.

### Leaf gradient expression data

Publicly available RNA-Seq data of leaf gradients were mined from four C_4_ grasses and two C_3_ grasses (Supplementary Data Table S1). The TPM values were extracted as described above. The expression profiles were represented as mean log_2_ TPM values for at least three biological replicates (except for *S. viridis*, which had only one biological replicate). For each species, between 5 and 15 leaf sections were segmented and sampled for RNA-Seq analysis. These values can be found in Supplementary Data File S3.

### Bundle sheath and mesophyll cell expression data

RNA-Seq data were obtained from the bundle sheath and mesophyll cells of several C_4_ grasses (Supplementary Data Table S1). We extracted TPM values as described above. Each study had at least three biological replicates for both cell types. The expression of genes is represented as TPM compared between the bundle sheath and mesophyll cells for each species. The TPM values were also averaged across all the C_4_ grasses that were surveyed for each cell type. These values can be found in Supplementary Data File S4.

### Data analysis

Figures and statistical analyses were performed using GraphPad Prism v9.4.1. A paired Student’s *t*-test was used to compare the leaf-to-seed expression ratios of each sugar sensor gene to determine if it there was preferential expression to the source or sink tissue (Supplementary Data Table S2). A paired Student’s *t*-test was also carried out to compare the expression between bundle sheath and mesophyll cells of sugar sensor genes in C_4_ grasses (Supplementary Data Table S3).

## RESULTS

### Identification of genes encoding sugar sensor components in C_4_ and C_3_ grasses

Identification of putative sugar sensors across selected C_4_ and C_3_ grasses was first carried out using BLAST searches of the respective genomes using known sequences from either *Z. mays* or *Arabidopsis*. Extracted sequences were translated and phylogenetic trees built to visualize homology to each other (Supplementary Data Figs S1–S6). Sequences from *Z. mays*, *S. spontaneum*, *S. bicolor*, *S. viridis*, *S. italica*, *P. miliaceum*, *P. hallii*, *O. sativa* and *B. distachyon* were extracted from their respective genomes where present.

Table 1 summarizes the sugar sensor genes that were present or absent in each grass species. The *B. distachyon* genome did not contain a copy of *RAPTOR2* and some grasses did not have a copy of *RAPTOR3*. Several grasses, such as *Z. mays*, *P. miliaceum* and *S. spontaneum*, also contained a second hybrid subunit within their genomes (Supplementary Data Fig. S4). The number of hexokinase homologues varied between six and nine across the grasses analysed (Supplementary Data Fig. S5). Each genome contained a copy of *HXK5* and a *HXK6*, the putative sugar sensors. Each species also contained a copy of *TPS1*, *TPP* and *TRE* (Supplementary Data Fig. S6). Analysis of C_4_ and C_3_ grasses did not appear to show gene duplication during evolution.

**Table 1.** Sugar sensor genes present in C_4_ and C_3_ grasses. *Brachypodium distachyon* (Bd), *Oryza sativa* (Os), *Panicum hallii* (Ph), *Panicum miliaceum* (Pm) *Sorghum bicolor* (Sb), *Setaria italica* (Si), *Setaria viridis* (Sv), *Saccharum spontaneum* (Ss) and *Zea mays* (Zm). ‘X’ denotes the presence of the gene within the genome of the corresponding species.

Gene	Bd	Os	Zm	Pm	Sv	Ss	Sb	Si	Ph
*TOR*	X	X	X	X	X	X	X	X	X
*LST8-1*	X	X	X	X	X	X	X	X	X
*RAPTOR1*	X	X	X	X	X	X	X	X	X
*RAPTOR2*		X	X	X	X	X	X	X	X
*RAPTOR3*	X		X	X		X		X	
*SnRK1α1*	X	X	X	X	X	X	X	X	X
*SnRK1α2*	X	X	X	X	X	X	X	X	X
*SnRK1α3*	X	X	X	X	X	X	X	X	X
*SnRK1β1*	X	X	X	X	X	X	X	X	X
*SnRK1β2*	X	X	X	X	X	X	X	X	X
*SnRK1β3*	X	X	X	X	X	X	X	X	X
*SnRK1γ1*	X	X	X	X	X	X	X	X	X
*SnRK1γ2*	X	X	X	X	X	X	X	X	X
*SnRK1βγ1*	X	X	X	X	X	X	X	X	X
*SnRK1βγ2*			X	X		X			
*HXK1*		X							
*HXK2*	X	X							
*HXK3*	X	X	X	X	X	X	X	X	X
*HXK5*	X	X	X	X	X	X	X	X	X
*HXK6*	X	X	X	X	X	X	X	X	X
*HXK4*	X	X	X						
*HXK7*	X	X	X		X	X	X	X	X
*HXK8*	X		X	X	X	X	X	X	X
*HXK9*	X	X	X	X		X	X		
*HXK10*	X	X	X	X	X	X	X	X	X
*TPS1*	X	X	X	X	X	X	X	X	X
*TPP1*	X	X	X	X	X	X	X	X	X
*TRE*	X	X	X	X	X	X	X	X	X

Positive selection analyses were carried out for each putative sugar sensor using the extracted protein sequences to determine if there was any detectable C_4_-dependent evolution within the set of genes. Sequences used were from four C_4_ grasses, *P. antidotale*, *S. bicolor*, *S. viridis* and *Z. mays*, and five C_3_ grasses, *H. amplexicaulis*, *P. bisulcatum*, *S. laxum*, *O. sativa* and *C. patens*. Nicotinamide-adenine dinucleotide phosphate-malic enzyme (NADP-ME) was used as a control for these analyses as it is known that several residues of this gene are under C_4_-specific selection. As expected, several residues were identified as being under C_4_-specific selection in NADP-ME (Supplementary Data Fig. S7). However, no residues were identified as being under C_4_-specific selection within any of the sugar sensor proteins tested here (TOR, LST8-1, RAPTOR1, RAPTOR2, SnRK1α, β, γ and βγ subunits, HXK5, HXK6, TPS1, TPP1 and TRE).

### Sugar sensor genes are expressed in both the source and sink tissues of C_4_ and C_3_ grasses

**Fig. 1.**
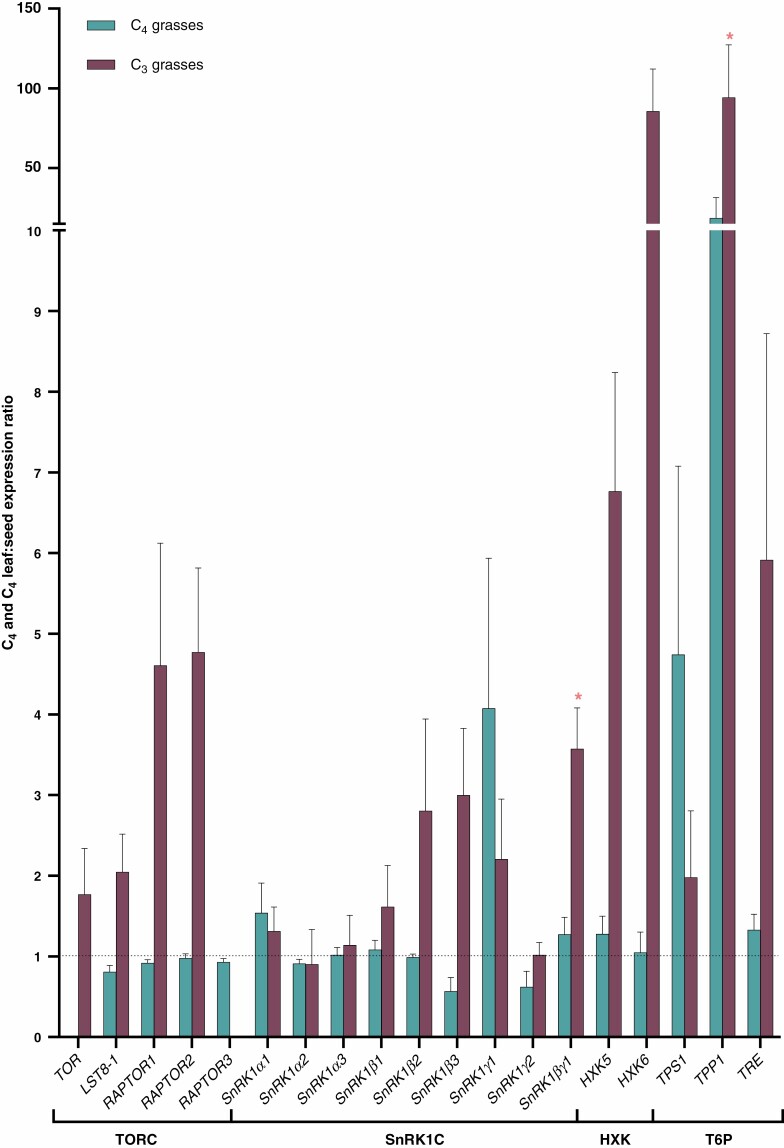
Leaf-to-seed expression ratio of sugar sensor gene comparisons between C_4_ and C_3_ grasses. RMA-normalized or TPM values from either microarray or RNA-Seq data of sugar sensor genes were used to calculate leaf-to-seed ratios from the C_4_ grasses *Z. mays* and *P. miliaceum* and the C_3_ grasses *O. sativa* and *B. distachyon* (Jain *et al.*, 2007; Sekhon *et al.*, 2011; Yue *et al.*, 2016; Sibout *et al.*, 2017). Each species consisted of three biological replicates for each tissue sampled. Data represent the mean leaf-to-seed ratios of genes from C_4_ and C_3_ grasses (*n* = 6). Error bars represent the standard error of the mean. Ratios <1 indicate expression of the gene predominating in the seed whereas ratios >1 indicate expression predominating in the leaf. The broken line indicates 1. Asterisks represent a significant difference from 1 and predominating in the leaf. There were no genes expressed with a ratio <1 that were significantly different.

Source (leaf) and sink (seed) transcriptomic data were scrutinized to determine whether differences at the gene expression level are associated with the evolution of C_4_ photosynthesis (Fig. 1, Supplementary Data File S1). Using publicly available leaf and seed transcriptomic data, gene expressions of sugar sensor genes were extracted for two C_4_ grasses (*Z. mays* and *P. miliaceum*) and two C_3_ grasses (*O. sativa* and *B. distachyon*) (Jain *et al.*, 2007; Sekhon *et al.*, 2011; Yue *et al.*, 2016; Sibout *et al.*, 2017). Data were combined for each photosynthetic type for analysis. Within these datasets, *TOR* was not in either of the datasets for the C_4_ grasses, and RAPTOR*3* expression was not detected in the C_3_ grass *B. distachyon*. Genes encoding the TORC subunits exhibited heightened transcript expression in the leaves (source tissue) of C_3_ grasses, when compared with the ratios exhibited by C_4_ grasses. The source-to-sink ratios for the C_4_ grasses were close to 1 for many of the sugar-sensing genes, suggesting they are expressed equally between leaf and seed tissues, at least for these grasses. Only the gene encoding the regulatory subunit of SnRK1C (*SnRK1βγ1*) and the *TPP1* gene (which encodes the trehalose phosphate phosphatase enzyme) had significantly higher (*P* ≤ 0.05) expression in the leaf compared with the seed for the C_3_ grasses. There was no significant expression of any gene for the C_4_ species or towards the seed (i.e. <1 and significantly different from 1). It can also be noted that *HXK5* source-to-sink ratio was ~5.6-fold higher for the C_3_ grasses than the C_4_ grasses, and this change was even more apparent for HXK6 gene expression, at an ~58.3-fold difference. However, these differences were not identified as significant.

There were more significant changes (*P* ≤ 0.05) within individual species that indicate some sugar sensor genes may be preferentially expressed in the source or sink tissues (Supplementary Data Fig. S8). Notably, many genes identified as significantly different in *Z. mays* had a ratio <1, indicating higher amounts of transcripts were identified in the sink tissues. Preferential expression to either the leaf or seed is more prominent within a species rather than collectively as a photosynthesis type (Supplementary Data Fig. S1).

### Sugar sensor genes are largely stably expressed along the leaf gradient of C_3_ and C_4_ grasses

**Fig. 2.**
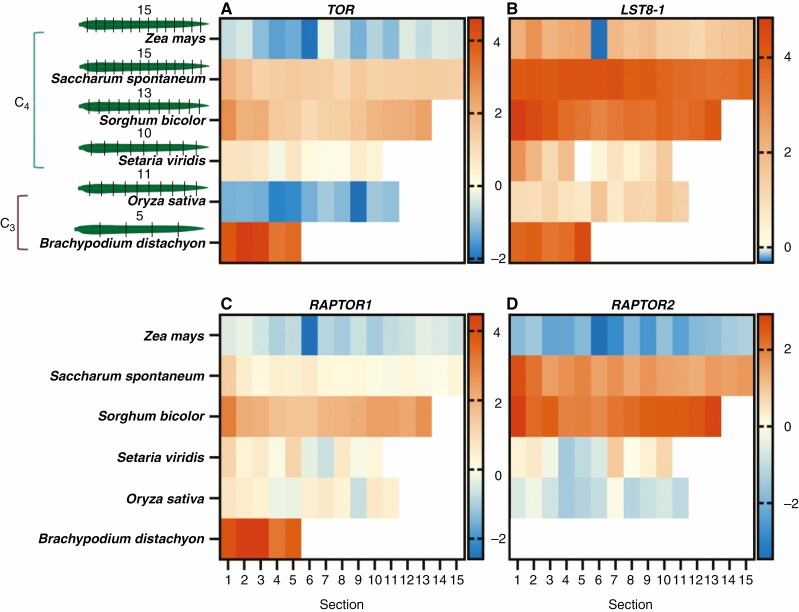
Expression of genes encoding TORC subunits along the leaf gradient of C_4_ and C_3_ grasses. Heat maps displaying log_2_ TPM values of the genes *TOR* (A), *LST8-1* (B), *RAPTOR1* (C) and *RAPTOR2* (D), encoding subunits that make up TORC. *RAPTOR3* is omitted due to its absence in multiple genomes. *RAPTOR2* was absent in the *Brachypodium* genome. The C_4_ species examined were *Z. mays* (15 sections), *Saccharum spontaneum* (15 sections), *Sorghum bicolor* (13 sections) and *Setaria viridis* (10 sections), the C_3_ species being *O. sativa* (11 sections) and *B. distachyon* (5 sections) (Li *et al.*, 2010; Wang *et al.*, 2014; Ding *et al.*, 2015; Hu *et al.*, 2018). Leaf sectioning is indicated to the left (A). Where expression within a leaf section is represented as white, there were no detectable reads. The scale bar to the right of each heat map represents log_2_ TPM.

**Fig. 3.**
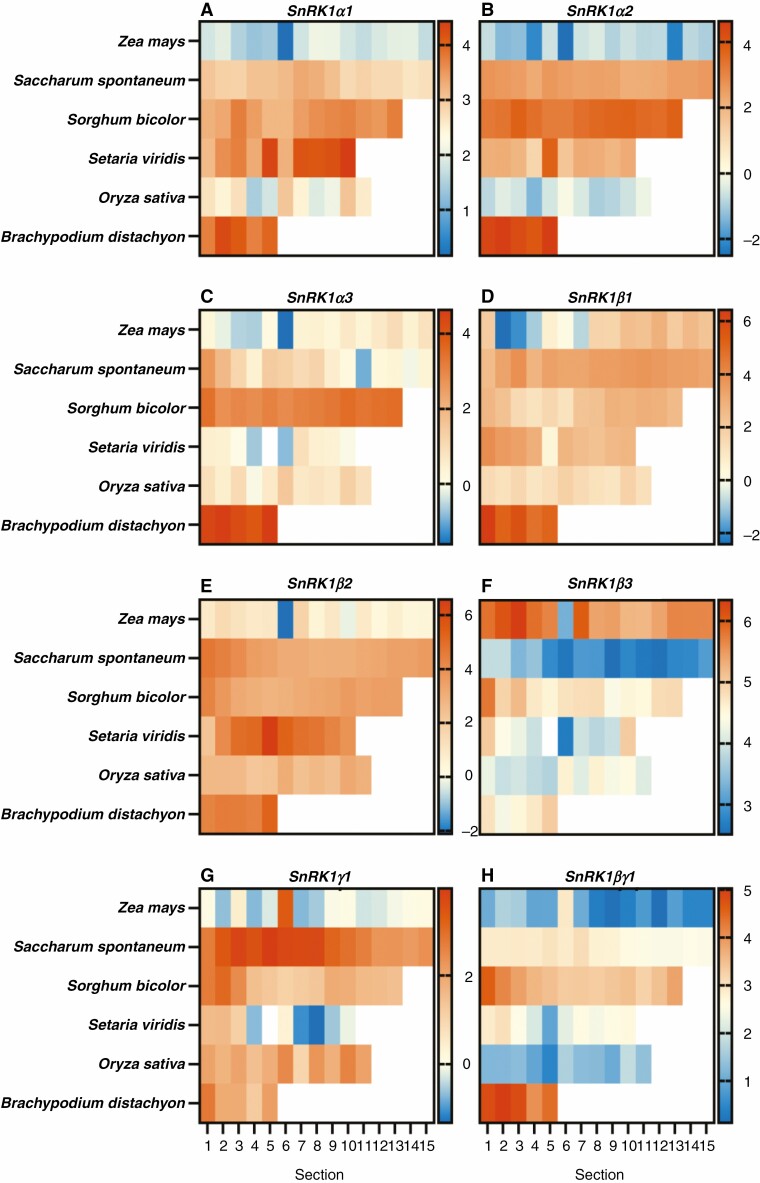
Expression of genes encoding SnRK1C subunits along the leaf gradient of C_4_ and C_3_ grasses. Heat maps displaying log_2_ TPM values of the genes *SnRK1α1* (A), *SnRK1α2* (B), *SnRK1α3* (C), *SnRK1β1* (D), *SnRK1β2* (E), *SnRK1β3* (F), *SnRK1γ1* (G) and SnRK1βγ1 (H), encoding subunits that make up SnRK1C. *SnRK1γ2* and *SnRK1βγ2* are omitted because of their absence in the genome or low to no expression within the species. The C_4_ species examined were *Z. mays* (15 sections), *Saccharum spontaneum* (15 sections), *Sorghum bicolor* (13 sections) and *Setaria viridis* (10 sections), the C_3_ species being *O. sativa* (11 sections) and *B. distachyon* (5 sections) (Li *et al.*, 2010; Wang *et al.*, 2014; Ding *et al.*, 2015; Hu *et al.*, 2018). Where expression within a leaf section is represented as white, there were no detectable reads. The scale bar to the right of each heat map represent log_2_ TPM.

**Fig. 4.**
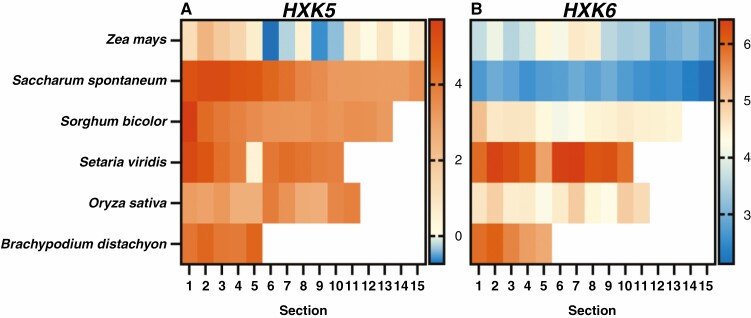
Expression of genes encoding putative sugar sensing hexokinases along the leaf gradient of C_4_ and C_3_ grasses. Heat maps displaying log_2_ TPM values of the genes *HXK5* (A), *HXK6* (B). The C_4_ species examined were *Z. mays* (15 sections), *Saccharum spontaneum* (15 sections), *Sorghum bicolor* (13 sections) and *Setaria viridis* (10 sections), the C_3_ species being *O. sativa* (11 sections) and *B. distachyon* (5 sections) (Li *et al.*, 2010; Wang *et al.*, 2014; Ding *et al.*, 2015; Hu *et al.*, 2018). Scale bar to the right of each heat map represent log_2_ TPM.

**Fig. 5.**
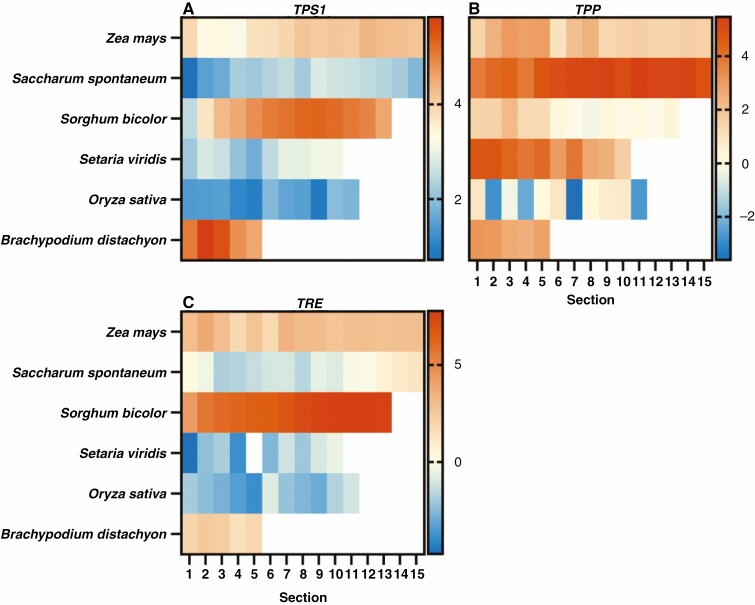
Expression of genes encoding proteins involved in T6P signalling along the leaf gradient of C_4_ and C_3_ grasses. Heat maps displaying log_2_ TPM values of the genes *TPS1* (A), *TPP* (B) and *TRE* (C). The C_4_ species examined were *Z. mays* (15 sections), *Saccharum spontaneum* (15 sections), *Sorghum bicolor* (13 sections) and *Setaria viridis* (10 sections), the C_3_ species being *O. sativa* (11 sections) and *B. distachyon* (5 sections) (Li *et al.*, 2010; Wang *et al.*, 2014; Ding *et al.*, 2015; Hu *et al.*, 2018). Where expression within a leaf section is represented as white there were no detectable reads. The scale bar to the right of each heat map represent log_2_ TPM.

Publicly available RNA-Seq data were mined for their expression along the leaf gradient of various C_3_ and C_4_ grasses and represented as heat maps (Li *et al.*, 2010; Wang *et al.*, 2014; Ding *et al.*, 2015; Hu *et al.*, 2018) (Figs 2–5). This analysis was carried out to determine whether there were possible changes in sugar sensing along the leaf and/or between C_4_ and C_3_ grasses during the sink-to-source transition from the base to the tip. The expression profiles for the genes encoding TORC subunits are displayed for *TOR*, *LST8-1*, *RAPTOR1* and *RAPTOR2* (Fig. 2A–D). *RAPTOR3* was excluded since this subunit was only found in some species. Notably, *B. distachyon* orthologues were expressed at high levels compared with the other grasses. Further, *LST8-1* and RAPTOR2 transcripts were also highly abundant along the leaf gradient in *S. spontaneum* and *S. bicolor*.

Similarly to genes that encode TORC subunits, the expression profiles of genes that encode the SnRK1C subunits were also examined along the leaf gradient of these grasses (Fig. 3). *SnRK1γ2* and *SnRK1βγ2* were excluded from the heat maps because they were absent in multiple genomes. The α subunits were found to be expressed in all species examined to varying levels (Fig. 3A–C). Expression was largely stable across the leaf in each species; however, some patterns were apparent. The genes encoding the α subunits of *S. bicolor* generally had higher expression towards the tip of the leaf. A similar pattern was observed for *SvSnRK1α1*. Conversely, *SsSnRK1β2* and *ZmSnRK1β3* had higher expression at the base of the leaf. In the middle sections of the leaf, *SvSnRK1β2* and *SsSnRK1γ1* were expressed at higher levels.

The expressions of the genes encoding putative sugar sensors, *HXK5* and *HXK6*, were examined to investigate whether glucose sensing may differ across the leaf gradient in C_4_ and C_3_ grasses (Fig. 4). *HXK5* was expressed at high levels for all species except for *Z. mays* (Fig. 4A). Notably, for several C_4_ grasses (*S. spontaneum*, *S. bicolor* and *S. viridis*) expression tended to be higher towards the base of the leaf for *HXK5*. Despite high homology between *HXK5* and *HXK6*, *HXK6* was largely expressed at low levels (Fig. 4B). Only S. *viridis* and *B. distachyon* exhibited high abundance of *HXK6* transcripts (Fig. 4B).

Finally, genes encoding enzymes associated with trehalose metabolism were interrogated along the leaf gradient. Similarly to many other genes encoding sugar sensing components, *TPS1*, *TPP* and *TRE* transcripts were expressed at varying degrees across the leaf and between species (Fig. 5). For *SbTPS1*, leaf sections eight to ten (around the middle) exhibited the highest transcript expression compared with the rest of the leaf (Fig. 5A). *SsTPP* was expressed relatively ubiquitously along the leaf at high levels, while *SvTPP* and *ZmTPP* were preferentially expressed at the base of the leaf (Fig. 5B). *SbTRE* was highly expressed when compared with the other grasses, especially towards the tip of the leaf (Fig. 5C).

### Sugar sensor genes exhibit species-specific preferential expression in either bundle sheath or mesophyll cells

**Fig. 6.**
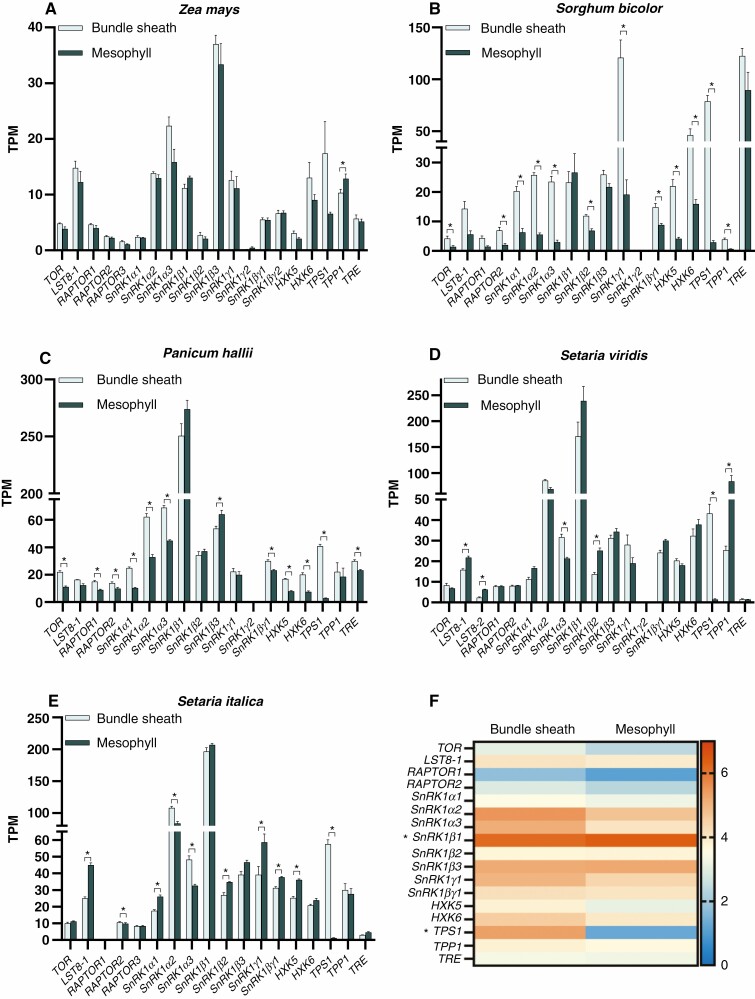
Sugar sensor gene expression between bundle sheath and mesophyll cells of C_4_ grasses: *Z. mays* (A), *Sorghum bicolor* (B), *Setaria viridis* (C), *Setaria italica* (D) and *P. hallii* (E) (John *et al.*, 2014; Döring *et al.*, 2016; Denton *et al.*, 2017; Washburn *et al.*, 2021). (F) Heat map comparison of log_2_ TPM means of bundle sheath and mesophyll expression in C_4_ grasses. *P < 0.05, significantly different expression between bundle sheath and mesophyll cells (Student’s *t*-test).

Transcript expression associated with sugar-sensing genes was analysed within bundle sheath and mesophyll cells of several C_4_ grasses from publicly available RNA-Seq datasets (John *et al.*, 2014; Döring *et al.*, 2016; Denton *et al.*, 2017; Washburn *et al.*, 2021). These results are presented for *Z. mays*, *S. bicolor*, *S. viridis*, *S. italica* and *P. hallii* (Fig. 6, Supplementary Data File S4, Supplementary Data Table S3). Datasets from C_3_ grasses were not examined in this study since they were sparse or had poor mapping of reads to their respective genomes, likely due to the difficulty of isolating and separating these cells in C_3_ species.

In *Z. mays*, sugar sensor genes were generally not preferentially expressed in one photosynthetic cell type (Fig. 6A). *ZmSnRK1β3* showed the highest overall expression out of all the genes surveyed, while *ZmTPP* expression was significantly higher (*P* ≤ 0.05) in the mesophyll cells. Unlike *Z. mays*, in *S. bicolor* and *P. hallii* there were numerous significant differences (*P* ≤ 0.05) in transcript expression of sugar sensor genes, with almost all those identified as significant being elevated in bundle sheath cells (Fig. 6B, C). All genes encoding the TORC subunits (except *PhLST8-1*) and SnRK1α were shown to be significantly preferentially expressed in the bundle sheath cells of *S. bicolor* and *P. hallii.* Where the two species differed was the transcript expression of *SnRK1β3*, which was expressed preferentially in mesophyll cells of *P. hallii*, but not *S. bicolor*. The largest fold changes were for *TPS1*, for which there was a 27- and 15-fold increase in bundle sheath cells in *S. bicolor* and *P. hallii*, respectively. *Setaria viridis* and *S. italica* are two close relatives within the millets. Both species exhibited less significant differences in sugar sensor gene expression between the two photosynthetic cell types when compared with *S. bicolor* (Fig. 6D, E). In addition, unlike *S. bicolor*, numerous genes had significantly higher (*P* ≤ 0.05) expression in mesophyll cells of both or one of the species. Transcript expression of LST8-1 was significantly elevated in mesophyll cells of both *S. viridis* and *S. italica*. Several genes encoding the SnRK1C subunits showed significant differences in transcript expression between the two cells. *SvSnRK1α3* and *SvSnRK1β2* showed preference for the bundle sheath and mesophyll, respectively. *SiSnRK1α1* exhibited significantly elevated expressed in the mesophyll cells, although this was reversed for the other two *SiSnRK1α* subunit genes. Similar to *S. bicolor* and *P. halli*, there were also significant ~29.7- and ~47.3-fold increases in expression of *TPS1* in bundle sheath cells of *S. viridis* and *S. italica*, respectively.

The values for all sugar sensor genes examined were averaged and the resulting log_2_ TPM values were visualized for each cell type (Fig. 6F). Within this analysis, only *SnRK1β1* and *TPS1* transcript expressions were significantly different between the bundle sheath and mesophyll cells (Supplementary Data Table S3). The average *SnRK1β1* expression was higher in mesophyll cells for the C_4_ grasses examined; however, the fold differences were small. Although not significant, generally there was higher transcript abundance within the bundle sheath cell for *SnRK1α2*, *SnRK1α3*, *SnRK1γ1*, *HXK5* and *HXK6*. Like with the source-to-sink expression comparison, significant changes within a species were more common than significant changes associated with photosynthetic type.

## DISCUSSION

### No co-optional evolution for C_4_ sugar sensors but some species-specific preferential expression in bundle sheath or mesophyll cells

In this study it was hypothesized that sugar sensors have evolved to accommodate the two-celled compartmentation of C_4_ photosynthesis. To determine whether sugar sensors diverged from their C_3_ counterparts during the evolutionary transition from C_3_ to C_4_ photosynthesis, transcript sequences from C_4_ species (*P. antidotale*, *S. bicolor*, *S. viridis* and *Z. mays*) and C_3_ species (*H. amplexicaulis*, *P. bisulcatum*, *S. laxum*, *O. sativa* and *C. patens*) were utilized. No evidence for the positive selection of C_4_ sugar sensors during C_4_ evolution was identified within this study. This result was unexpected, as the evolution of C_4_ photosynthesis has resulted in major changes, involving C_4_-specific residue changes in numerous key genes (Christin *et al.*, 2009; Watson-Lazowski *et al.*, 2018). However, it is plausible that selection pressures associated with C_4_ photosynthesis have not influenced the sugar sensors in this specific way. For example, it has been well established that gene duplications have occurred during the evolution of C_4_ photosynthesis but it was not observed for genes in this study (Marshall *et al.*, 1996; Monson, 1999, 2003). Moreover, changes to *cis*-regulatory elements in single-copy genes have contributed to the altered expression patterns that facilitate C_4_ photosynthesis (Rosche and Westhoff, 1995). Aspects such as these may still facilitate C_4_-specific expression patterns of sugar-sensing genes.

When examining the general expression of the genes that encode the proteins that make up TORC there was little change between the bundle sheath and mesophyll cells of the C_4_ grasses (Fig. 6). The expression of TOR varied between species and was not significantly expressed in one cell type over another when collectively examining the C_4_ grasses, which suggests that the TOR protein has a signalling role in both cells. Studies in the alga *Chlamydomonas reinhardtii* have shown that CO_2_ fixation promotes TOR activity but has no effect on TOR or LST8 protein abundance (Mallen-Ponce *et al.*, 2022). Furthermore, it was observed that photosynthesis inhibition decreases TOR activity. The variation in gene expression for the subunits that encode TORC could be also related to the role it has in the circadian rhythm (Xiong and Sheen, 2014; Dong *et al.*, 2017). Therefore, tissue harvest time across studies could influence transcript abundance of the genes encoding TORC subunits. Moreover, its role as a master regulator across different tissues and processes could also account for the lack of differences in gene expression between the two photosynthetic cell types when collectively analysing the C_4_ grasses in this study (Pacheco *et al.*, 2021). Sucrose and starch synthesis occurs in the mesophyll and bundle sheath cells of C_4_ species, respectively. Although TORC is regulated by sugars, the complex also regulates starch accumulation. These observations could account for the presence of genes relating to this complex in both cell types.

Like TORC, SnRK1C is thought to regulate many processes, and is usually upregulated under stress conditions when sucrose availability is low (Baena-González *et al.*, 2007). Although the data were generated from plants grown in normal conditions, the overall transcript abundance of genes encoding SnRK1C subunits was high in comparison with the other sugar sensors (Fig. 6). On average, within the C_4_ grasses examined, *SnRK1β1* was preferentially expressed in mesophyll cells (Fig. 6F, Supplementary Data Table S3). *SnRK1β1* encodes a regulatory component of the complex and β subunits can be expressed at varying levels depending on the tissue, developmental stage and environmental cues (Polge *et al.*, 2008). Therefore, it is a possibility that SnRK1β1 regulates the interaction of the kinase with its targets within the mesophyll cells of C_4_ grasses. When averaged across the C_4_ species surveyed, *SnRK1α* genes (which encode the catalytic subunit of the complex) were not significantly different between cells. Nevertheless, it must be noted that many of the grasses had higher expression within the bundle sheath cells of the *SnRK1α* catalytic subunit genes (Fig. 6B–E). There is a possibility that SnRK1α subunits are important for sensing and/or signalling during sucrose translocation, in which photoassimilates pass through the bundle sheath cells for phloem loading to occur (Bezrutczyk *et al.*, 2018; Chen *et al.*, 2022). During this process, genes encoding regulatory subunits may be expressed according to translocation needs and photosynthetic activity. Alternatively, expression in the bundle sheath cells could be linked to a role in regulating genes associated with starch synthesis, which has been evidenced in the seed (Zhang *et al.*, 2001; Tiessen *et al.*, 2003).

As mentioned previously, T6P signalling has been closely linked with SnRK1C activity (Baena-González and Lunn, 2020). *TPS1* expression was significantly higher in the bundle sheath cells when averaged across the C_4_ grasses (Fig. 6F). TPS is involved in the synthesis of T6P, and its presence indicates elevated sucrose levels (Grennan, 2007). Therefore, it was surprising that *TPS1* was higher in the bundle sheath cells, since sucrose biosynthesis occurs predominantly in the mesophyll cells of C_4_ grasses (Lunn and Furbank, 1999; Furbank and Kelly, 2021). As suggested with SnRK1C, T6P signalling may play an important role in the phloem loading process (Emms *et al.*, 2016; Bezrutczyk *et al.*, 2018, 2021; Chen *et al.*, 2022). Interestingly, trehalose increases the expression of *ApL3*, which encodes an ADP-glucose pyrophosphorylase that subsequently increases starch synthesis (Wingler *et al.*, 2000). Therefore, the trehalose biosynthesis pathway may be important for starch production within the bundle sheath cells of C_4_ grasses. The additional genes encoding enzymes associated with T6P metabolism were expressed at similar levels between the bundle sheath and mesophyll cells for all species analysed. This could suggest that the synthesis, breakdown and signalling of trehalose is important in both cells, or, since trehalose is a non-reducing disaccharide, it could also play a role in buffering sucrose loading into the phloem.

There were also differences in expression of the putative HXK sugar sensors, *HXK5* and *HXK6*, between the two photosynthetic cells for several C_4_ species. For example, *SbHXK5*, *SbHXK6*, *PhHXK5* and *PhHXK6* were all expressed at higher levels in the bundle sheath compared with mesophyll cells (Fig. 6B, C). This could indicate that the phosphorylation of glucose is more prevalent in the bundle sheath cells of C_4_ species, or that glucose sensing predominates there. Research on the effect of HXK sugar sensing in C_4_ species has been sparse, but seminal studies using *Z. mays* protoplasts have shown that glucose, the substrate for HXK, can repress photosynthesis genes (Sheen, 1990; Jang and Sheen, 1994). It must be noted that this was only examined in a single-cell system and did not examine the whole leaf or how the plant that might affect how sugar sensing occurs, given photosynthesis takes place in a two-cell system in *Z. mays*.

### Expression of sugar sensor genes changes in source–sink developmental models

To investigate the expression patterns of genes encoding sugar sensor components in source and sink tissues, we interrogated leaf and seed tissues as well as developmental leaf gradients to determine if there was preferential expression in the source or sink tissue. Given that source-to-sink expression gradients have been observed with sugar transporters and other genes associated with sugar metabolism in C_4_ grasses, it may be expected that similar expression patterns will be found for genes encoding the sugar sensor proteins (Bezrutczyk *et al.*, 2018; Hu *et al.*, 2018; Chen *et al.*, 2022).

As previously established, TORC is a master regulator of many different processes in the plant. Therefore, genes encoding this complex would more likely be found in all tissue types. For the C_4_ species examined, gene expression of the regulatory subunits of TORC were close to 1, whereas for the C_3_ species the expression of numerous TORC genes trended towards source tissue (ratio of <1) (Fig. 1). The expression of these genes was also ubiquitous along the leaf gradient of the four C_4_ grasses and the two C_3_ grasses (Fig. 2). Interestingly, it has been shown that when TORC repression is initiated, *S. viridis* showed a milder phenotype and a smaller magnitude of changes relating to primary metabolites and global gene expression when compared with the C_3_*Arabidopsis* (da Silva *et al.*, 2021). This might suggest that plant growth in C_4_ species is less rigorously controlled by TORC, or is less sensitive to changes in carbon status. Previous work on the relationship between CO_2_ fixation and TOR activity has suggested that CO_2_ fixation status can influence TOR activity, but not necessarily change the protein abundance (Mallen-Ponce *et al.*, 2022). Thus, this might also mean that the transcript abundance of the subunits of TORC may not change between source and sink tissues, but rather activity is modulated via other factors.

Like TORC, SnRK1C is thought to regulate numerous processes throughout the plant. The seed-to-leaf expression ratios of genes encoding the catalytic subunits of SnRK1C for both C_4_ and C_3_ species were close to 1, demonstrating that they are found in both the seed and leaf tissues of the analysed grasses (Fig. 1). This suggests that these genes play a similar role within the plant regardless of whether they are C_4_ or C_3_ species. When examining the expression of genes that encode subunits of SnRK1C over a leaf gradient, again there were no immediate trends that differentiate source to sink comparisons between the leaf and seed or within C_4_ and C_3_ leaves along the leaf gradient (Fig. 3). These data would suggests that SnRK1 is largely equally distributed across source and sink tissue of C_3_ and C_4_ grasses. However, SnRK1C is known to be activated in response to unfavourable conditions or during a starvation response (Baena-González *et al.*, 2007). Therefore, the lack of differences between source and sink tissues might not be uncommon since these plants were grown in normal conditions. In addition, there is evidence that SnRK1α is regulated on the post-transcriptional level (Lu *et al.*, 2007), which may also explain the limited differences identified.

While the link between photosynthesis and SnRK1C is not well documented, a direct link between HXK sugar sensing and modulating photosynthesis gene expression has been identified. This was first established using *Z. mays* protoplasts, as mentioned previously (Sheen, 1990; Jang and Sheen, 1994). This was later confirmed using *gin2* mutants of *Arabidopsis*, showing AtHXK1 could sense glucose and in turn influence photosynthesis gene expression (Moore *et al.*, 2003). Sugar sensors have also been established in rice (C_3_ grass) via overexpression lines of OsHXK5 and OsHXK6, which exhibited heightened sensitivity to glucose (Cho *et al.*, 2006; Cho *et al.*, 2009*b*). These rice lines were generally smaller than wild-type and showed decreased expression of key photosynthesis genes, such as the Rubisco small subunit gene (*rbcS*). The homologues of *HXK5* and *HXK6* were expressed in both the leaf and seed tissues of the C_4_ and C_3_ species examined (Fig. 1). The source-to-sink ratio for C_4_ grasses was close to 1, whereas for C_3_ grasses it was well above 1. Although these differences were not significant within our dataset, the extent of the differences would suggest that HXKs predominate in the leaves of C_3_ grasses. Expression of *HXK5* and *HXK6* homologues was apparent all along the leaves of both C_4_ and C_3_ grasses, and changes along the leaf were subtle (Fig. 4). This is unlike sugar transporters and starch and sugar metabolism genes, which exhibited a more prominent gradient as the tissue changes from sink to source from the base to the tip of the leaf (Chen *et al.*, 2022). For *HXK5*, there was higher abundance at the base of the leaf, where it is more sink-like tissue, for the C_4_ grasses *S. spontaneum*, *S. bicolor* and *S. viridis* (Fig. 4A). This may suggest a role in sensing incoming photoassimilates that break down to glucose for utilization as the tissue matures. However, like TOR and SnRK1α, HXK sugar sensors could also be post-transcriptionally regulated, and so changes in expression may be minor and not correlate with activity.

T6P abundance is thought to modulate SnRK1C activity, subsequently de-repressing anabolic processes (Baena-González *et al.*, 2007; Lawlor and Paul, 2014). SnRK1C is known to be involved in starch synthesis during grain filling of grasses by regulating the expression of genes encoding proteins involved in this process, and there have also been suggestions that its activity is controlled by T6P levels (Laurie *et al.*, 2003; Lu *et al.*, 2007; Gazzarrini and Tsai, 2014). In this study, the *TPP1* source-to-sink expression ratio was significantly above 1 for C_3_ grasses, which may suggest that T6P (or trehalose itself) have a larger role in sugar sensing and signalling within the leaves (Fig. 1). The transgenic manipulation of *TPP1* in *Z. mays* showed that T6P plays a large role in coordinating photoassimilate partitioning to the reproductive tissues by regulating photosynthesis (Oszvald *et al.*, 2018). The authors showed that *Sugars Will Eventually be Exported Transporters* (*SWEET*) genes were upregulated in the transgenic lines, increasing the movement of photoassimilates to sink tissue, particularly under drought conditions. Other genes associated with T6P metabolism also had source-to-sink ratios above 1 in this study, although these differences were not significant. Further analysis on the expression of these genes along the leaf gradient of C_4_ and C_3_ grasses showed that they were expressed throughout, and many only showed small changes from the base to the tip (Fig. 5).

### Conclusions

In this study, transcriptomic data across various C_4_ and C_3_ grasses were analysed to determine gene expression patterns of the components of TORC, SnRK1C and HXK sugar sensors, as well as T6P metabolism. These analyses focused on the role of these sugar sensors in relation to photosynthesis, where sugars are produced, and whether sugars may be perceived differently between C_4_ and C_3_ grasses. Even though C_3_ grasses perform photosynthetic and carbohydrate production reactions in one cell type, unlike C_4_ grasses, which is compartmentalized, not many changes in sugar sensor gene expression were observed between the two types of plants.

There were few distinct gradient transitions of expression for sugar sensor genes, suggesting sugar sensing is important along the whole young leaf. Moreover, when expression was examined in the two photosynthetic cell types in C_4_ grass leaves only, *SnRK1β1* and *TPS1* were preferentially expressed in the mesophyll and bundle sheath cells, respectively. However, it must be noted that within species there were more distinct changes in expression of each sugar sensor gene.

Future studies could be incorporated to analyse sugar sensors between C_4_ and C_3_ grasses by examining protein abundance and activity to determine if sugars are perceived differently. These studies can also be expanded into a larger variety of C_4_ and C_3_ species that include dicots and monocots and different subtypes of C_4_ photosynthesis. Nevertheless, this study provides a foundation for which the role of sugar sensors can be scrutinized, especially in terms of how it may relate to C_4_ and C_3_ photosynthesis.

## Supplementary Material

mcad057_suppl_Supplementary_Data

mcad057_suppl_Supplementary_Material

## SUPPLEMENTARY DATA

Supplementary data are available online at https://academic.oup.com/aob and consist of the following. Figure S1: phylogenetic tree of monocot TOR complex subunits. Figure S2: phylogenetic tree of monocot SnRK1α subunits. Figure S3: phylogenetic tree of monocot SnRK1β subunits. Figure S4: phylogenetic tree of monocot SnRK1βγ and SnRK1γ subunits.

Figure S5: phylogenetic tree of monocot hexokinases. Figure S6: phylogenetic tree of monocot proteins related to T6P metabolism. Figure S7: C_4_-dependent evolution of NADP-ME. Figure S8: leaf-to-seed expression ratio of sugar sensor genes in C_4_ and C_3_ grasses. Table S1: summary of species used in this study, data accession numbers and references. Table S2: leaf-to-seed expression ratio of sugar sensor genes in C_4_ and C_3_ grasses. Table S3: bundle sheath and mesophyll cell sugar sensor gene expression in C_4_ grasses. File S1: gene IDs. File S2: leaf-to-seed expression ratios of C_4_ and C_3_ grasses. File S3: leaf gradient expression of sugar sensors from C_4_ and C_3_ grasses. File S4: bundle sheath and mesophyll expression of sugar sensors from C_4_ and C_3_ grasses.
