# Supplementary material for: Spatial expression patterns of genes encoding sugar sensors in leaves of C4 and C3 grasses
Source: Ann Bot. 2023 Apr 27;131(6):985–1000. doi: 10.1093/aob/mcad057 (PMC10332396; doi:10.1093/aob/mcad057)
Supplement: mcad057_suppl_Supplementary_Material [file mcad057_suppl_supplementary_material.docx]

**Table S1 Summary of species used in this study, data accession numbers and references.** Accession numbers can be used to search for raw data at either https://www.ncbi.nlm.nih.gov/ or https://www.ebi.ac.uk/. Bundle sheath cell (BSC), mesophyll cell (MC).

| **Species name** | **Photosynthetic pathway** | **Evolution** | **Leaf / Seed** | **Leaf gradient** | **BSC / MC** | **Data accession no.** | **Reference** |
| --- | --- | --- | --- | --- | --- | --- | --- |
| *Panicum antidotale* | C_4_ | X |  |  |  | PRJNA757558 | (Hussain et al., 2020) |
| *Panicum miliaceum* | C_4_ | X | X |  |  | SAMN05255231 | (Yue et al., 2016) |
| *Zea mays* | C_4_ |  | X |  |  | E-GEOD-27004 | (Sekhon et al., 2011) |
| *Zea mays* | C_4_ |  |  | X |  | PRJNA190188 | (Wang et al., 2014; Li et al., 2010) |
| *Zea mays* | C_4_ |  |  |  | X | PRJNA273547 | (Denton et al., 2017) |
| *Sorghum bicolor* | C_4_ |  |  | X |  | PRJNA275620 | (Ding et al., 2015) |
| *Sorghum bicolor* | C_4_ |  |  |  | X | PRJEB11652 | (Döring et al., 2016) |
| *Setaria viridis* | C_4_ |  |  | X |  | PRJNA279378 | (Ding et al., 2015) |
| *Setaria viridis* | C_4_ |  |  |  | X | PRJEB5074 | (John et al., 2014) |
| *Saccharum spontaneum* | C_4_ |  |  | X |  | PRJNA685968 | (Hu et al., 2018) |
| *Setaria italica* | C_4_ |  |  |  | X | PRJNA475365 | (Washburn et al., 2021) |
| *Panicum hallii* | C_4_ |  |  |  | X | PRJNA475365 | (Washburn et al., 2021) |
| *Steinchisma laxum* | C_3_ | X |  |  |  | PRJNA328851 | (Burke et al., 2016) |
| *Hymenachne amplexicaulis* | C_3_ | X |  |  |  | PRJNA395007 | (Moreno-Villena et al., 2018) |
| *Cyrtococcum patens* | C_3_ | X |  |  |  | PRJNA395007 | (Moreno-Villena et al., 2018) |
| *Panicum bisulcatum* | C_3_ | X |  |  |  | PRJNA392207 | (Watson-Lazowski et al., 2018) |
| *Oryza sativa* | C_3_ |  | X |  |  | GSE6893 | (Jain et al., 2007) |
| *Oryza sativa* | C_3_ |  |  | X |  | PRJNA190188 | (Wang et al., 2014) |
| *Brachypodium distachyon* | C_3_ |  | X |  |  | E-MTAB-5491 | (Sibout et al., 2017) |
| *Brachypodium distachyon* | C_3_ |  |  | X |  | PRJNA372178; PRJNA372180; PRJNA372182; PRJNA372183; PRJNA372185 | No reference, as part of the DOE JGI Plant Flagship Gene Atlas |

**
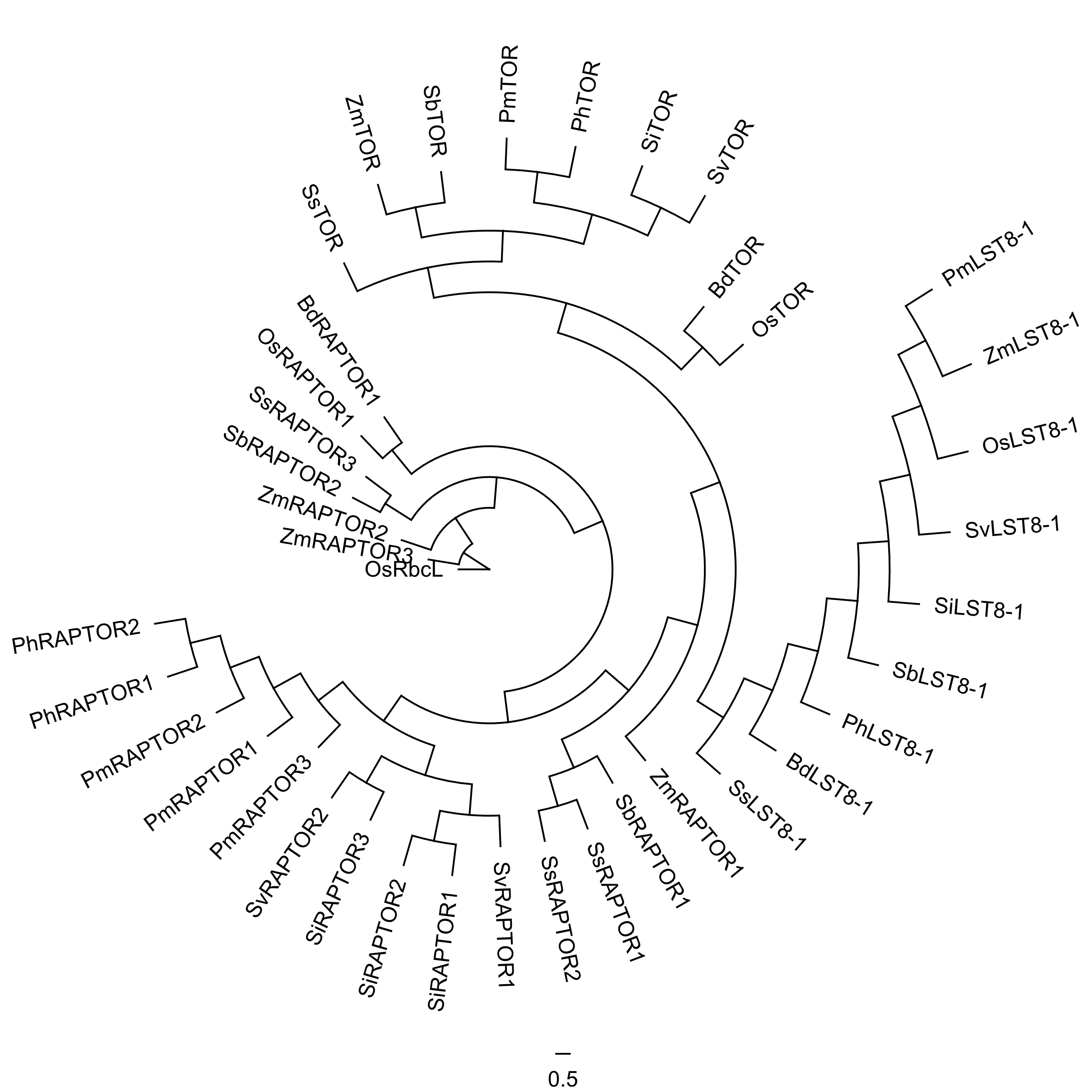
Figure S1 Phylogenetic tree of monocot TOR complex subunits.**

Phylogenetic tree constructed with Target of Rapamycin (TOR), Regulatory-Associated Protein of TOR 1 (RAPTOR), and Lethal with Sec Thirteen 8 (LST8) protein sequences from *Brachypodium distachyon* (Bd), *Oryza sativa* (Os), *Panicum* *hallii* (Ph), *Panicum* *miliaceum* (Pm) *Sorghum bicolor* (Sb), *Setaria italica* (Si), *Setaria viridis* (Sv), *Saccharum spontaneum* (Ss), and *Zea mays* (Zm). The tree is rooted with *Oryza sativa* Rubisco large subunit (OsRbcL). Branch lengths represent substitutions per site of the alignment sequence.

**Figure S2 Phylogenetic tree of monocot SnRK1α subunits.**


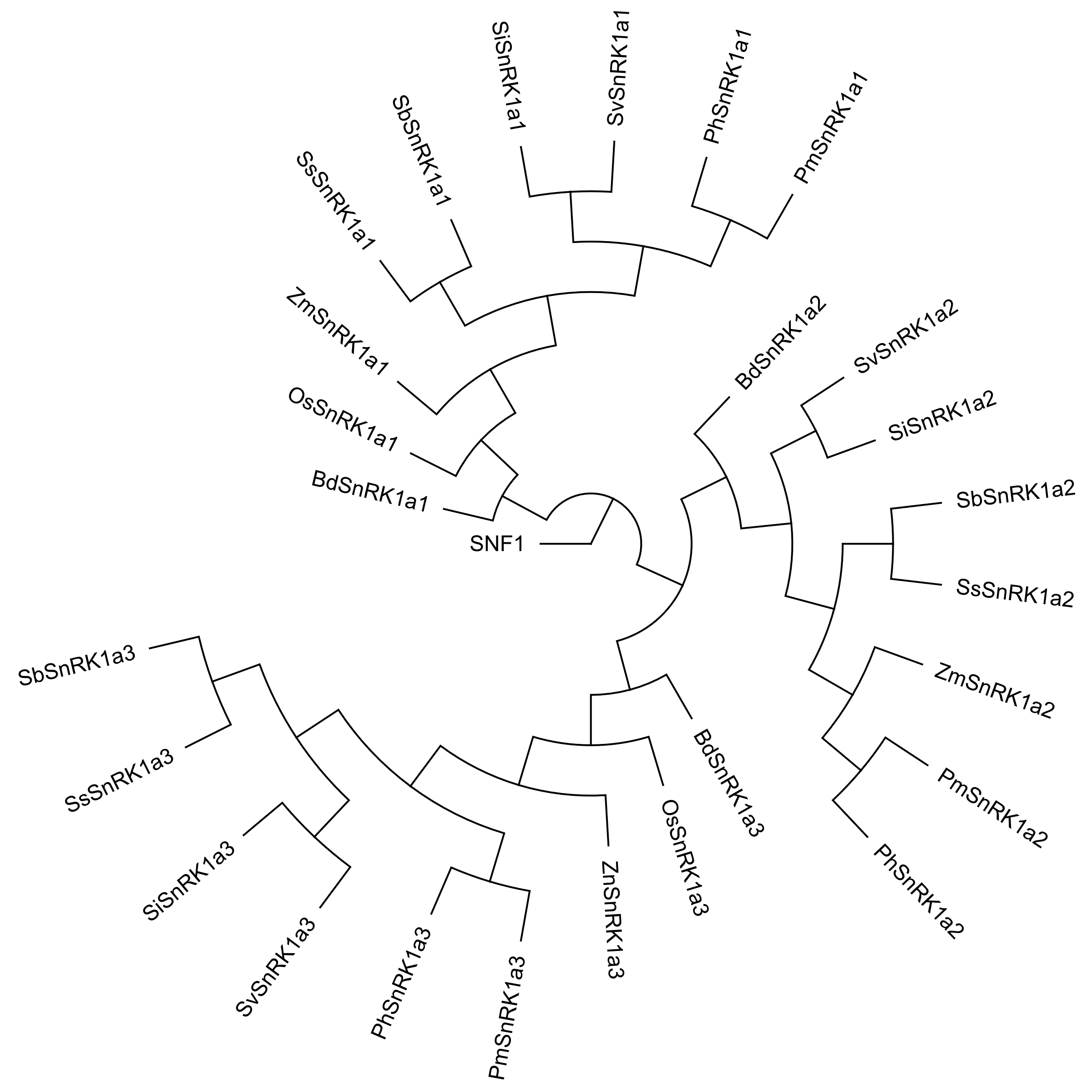

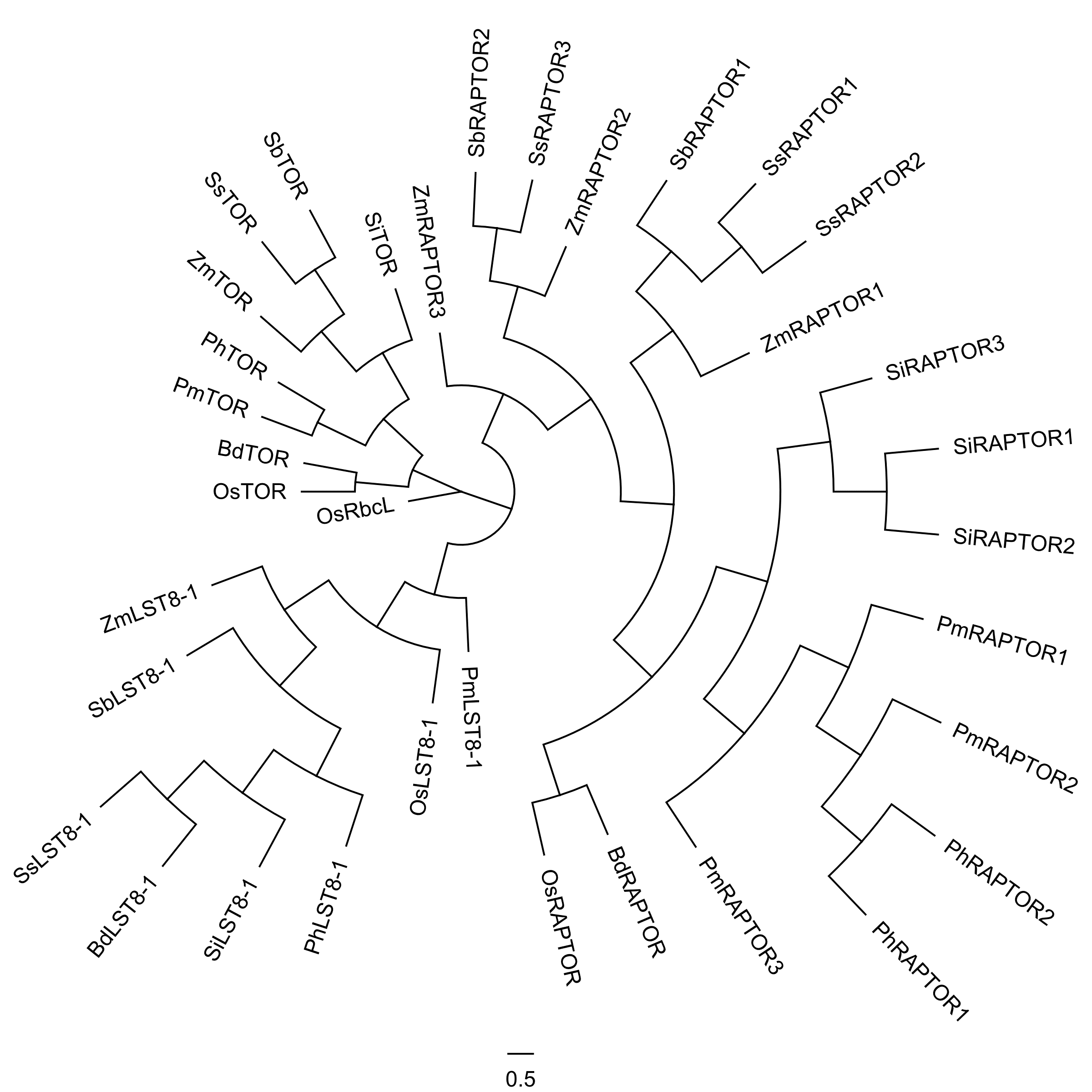


Phylogenetic tree constructed with Sucrose Non-Fermenting 1-related kinase 1 (SnRK1) α subunit protein sequences from *Brachypodium distachyon* (Bd), *Oryza sativa* (Os), *Panicum* *hallii* (Ph), *Panicum* *miliaceum* (Pm) *Sorghum bicolor* (Sb), *Setaria italica* (Si), *Setaria viridis* (Sv), *Saccharum spontaneum* (Ss), and *Zea mays* (Zm). The tree is rooted with yeast SNF1. Branch lengths represent substitutions per site of the alignment sequence.

**
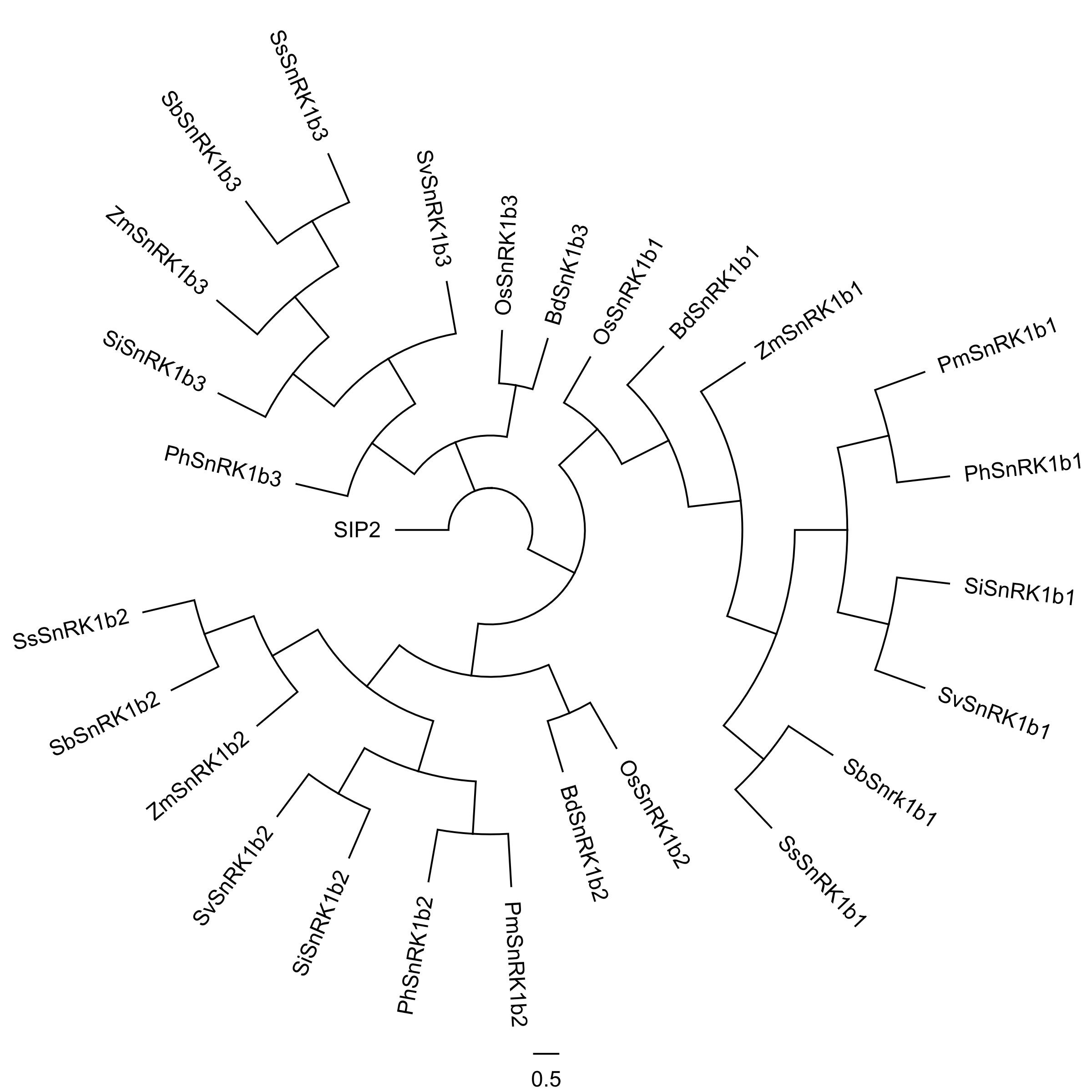
Figure S3 Phylogenetic tree of monocot SnRK1β subunits.**

Phylogenetic tree constructed with Sucrose Non-Fermenting 1-related kinase 1 (SnRK1) β subunit protein sequences from *Brachypodium distachyon* (Bd), *Oryza sativa* (Os), *Panicum* *hallii* (Ph), *Panicum* *miliaceum* (Pm) *Sorghum bicolor* (Sb), *Setaria italica* (Si), *Setaria viridis* (Sv), *Saccharum spontaneum* (Ss), and *Zea mays* (Zm). The tree is rooted with yeast SNF beta subunit (SIP2). Branch lengths represent substitutions per site of the alignment sequence.


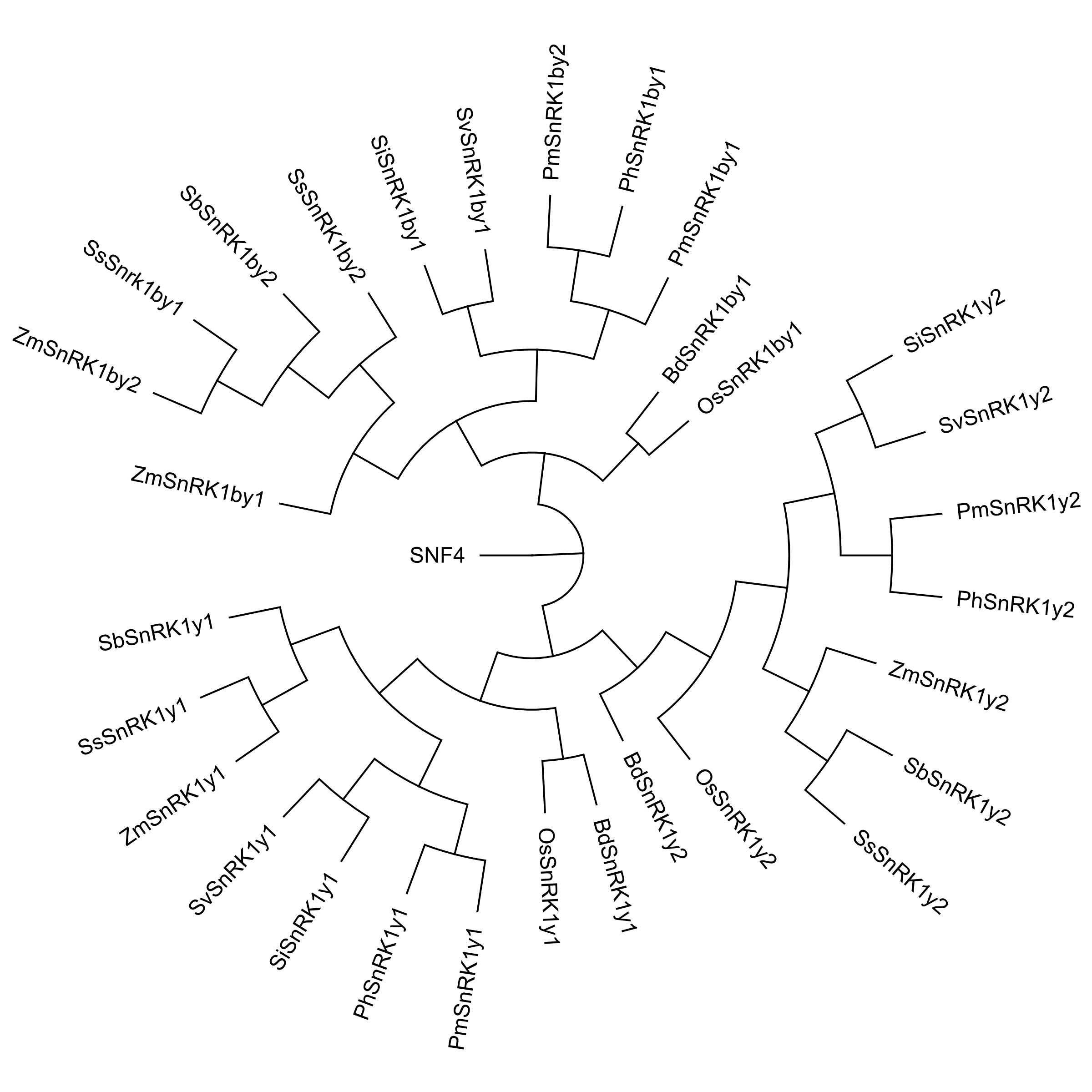

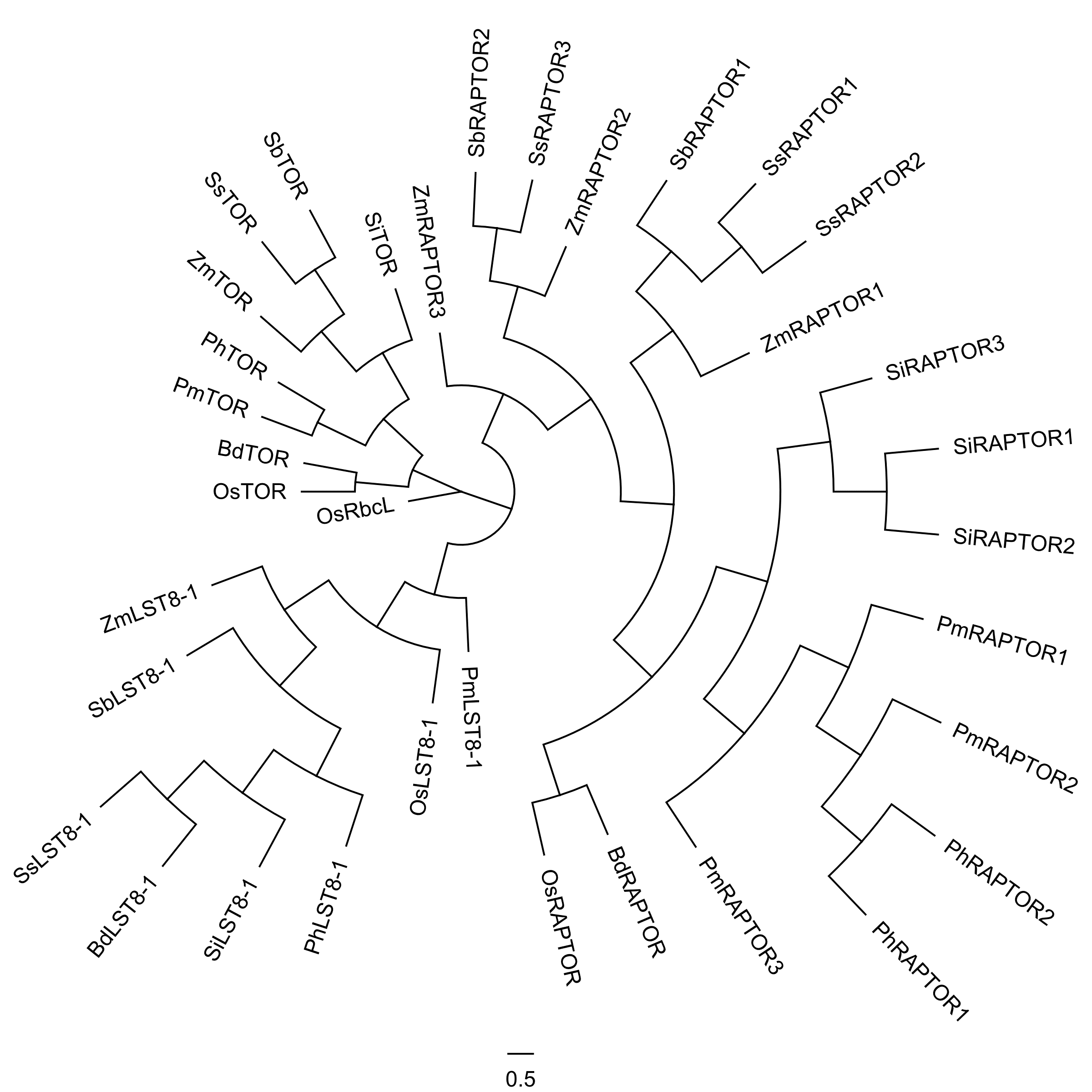


**Figure S4 Phylogenetic tree of monocot SnRK1βγ and SnRK1γ subunits.**

Phylogenetic tree constructed with Sucrose Non-Fermenting 1-related kinase 1 (SnRK1) βγ and SnRK1γ subunit protein sequences from *Brachypodium distachyon* (Bd), *Oryza sativa* (Os), *Panicum* *hallii* (Ph), *Panicum* *miliaceum* (Pm) *Sorghum bicolor* (Sb), *Setaria italica* (Si), *Setaria viridis* (Sv), *Saccharum spontaneum* (Ss), and *Zea mays* (Zm). The tree is rooted with yeast SNF beta gamma subunit (SNF4). Branch lengths represent substitutions per site of the alignment sequence.

**
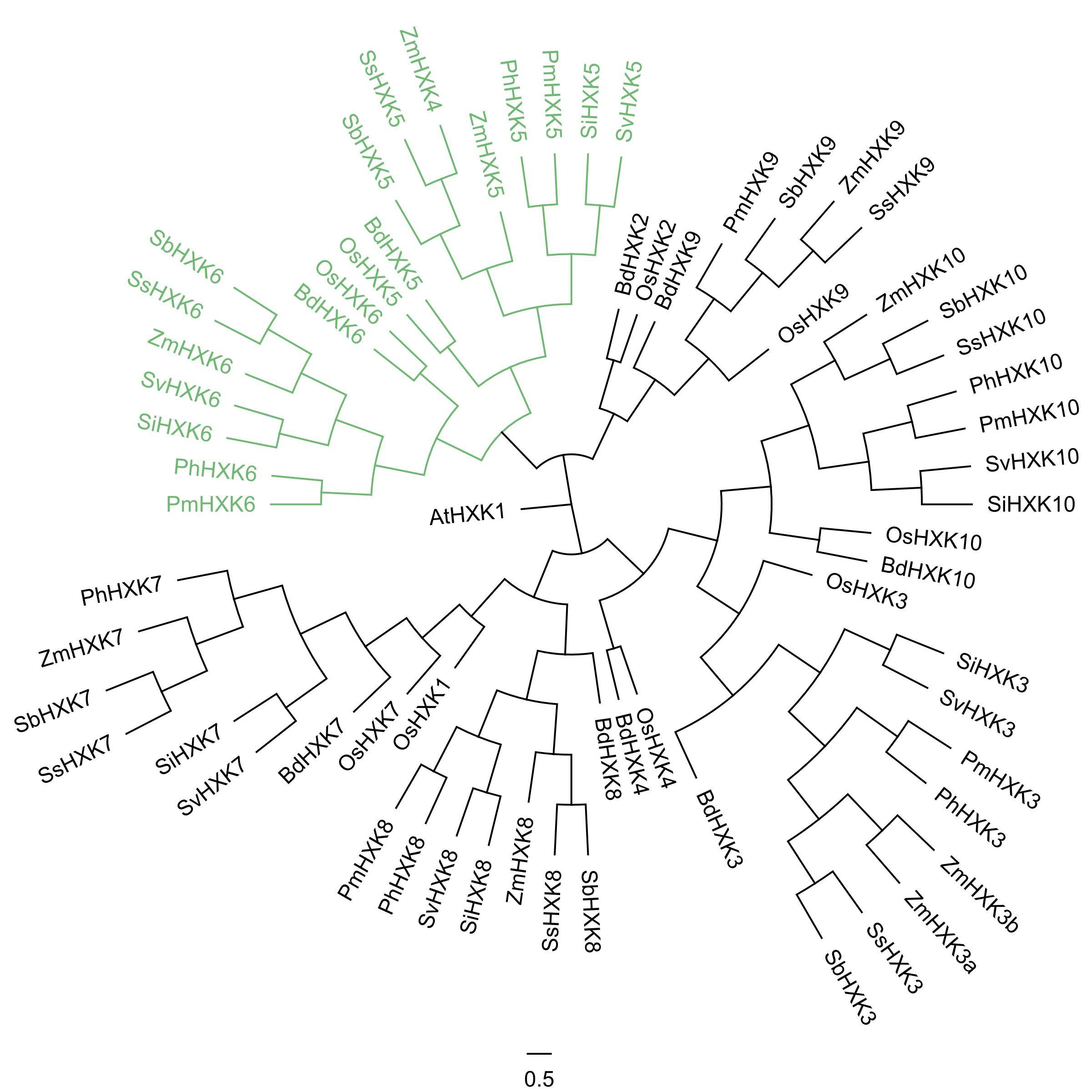
Figure S5 Phylogenetic tree of monocot hexokinases.**

Phylogenetic tree constructed with hexokinase (HXK) protein sequences from *Brachypodium distachyon* (Bd), *Oryza sativa* (Os), *Panicum* *hallii* (Ph), *Panicum* *miliaceum* (Pm) *Sorghum bicolor* (Sb), *Setaria italica* (Si), *Setaria viridis* (Sv), *Saccharum spontaneum* (Ss), and *Zea mays* (Zm). Putative sugar sensors are in green. The tree is rooted with Arabidopsis thaliana HXK1. Branch lengths represent substitutions per site of the alignment sequence.

**Figure S6 Phylogenetic tree of monocot proteins related to T6P metabolism.**


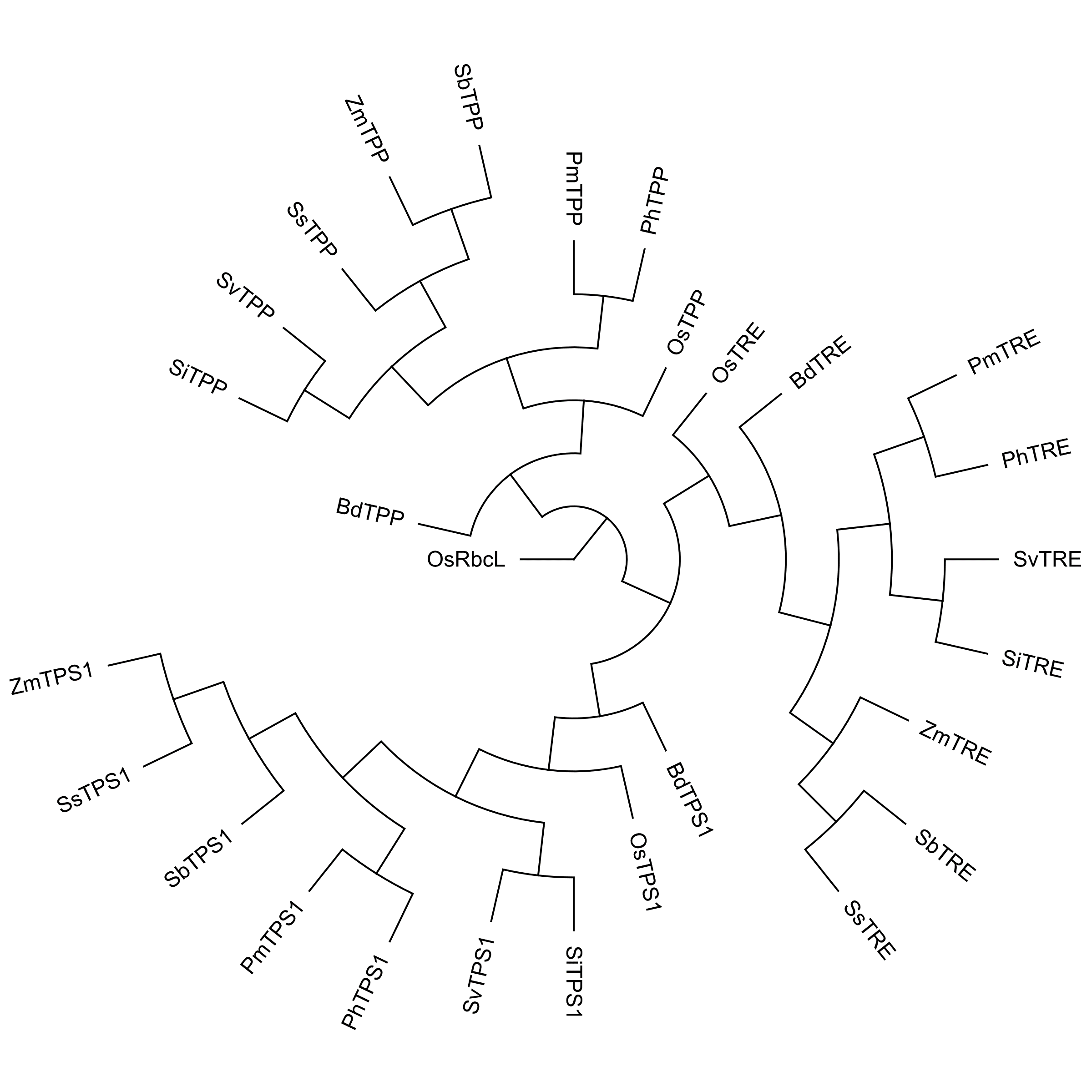

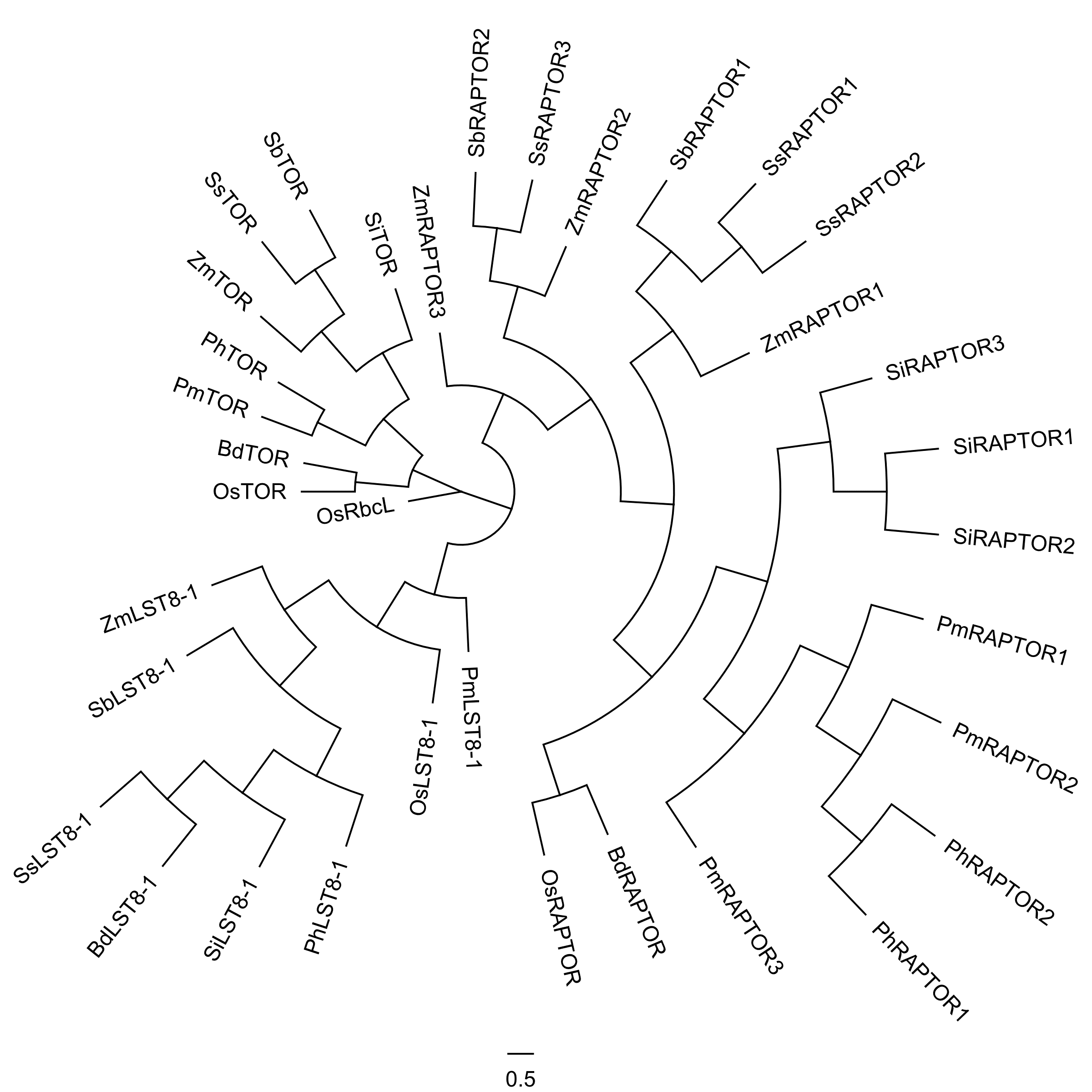


Phylogenetic tree constructed with trehalose phosphate synthase (TPS), trehalose phosphate phosphatase (TPP) and trehalase (TRE) protein sequences from *Brachypodium distachyon* (Bd), *Oryza sativa* (Os), *Panicum* *hallii* (Ph), *Panicum* *miliaceum* (Pm) *Sorghum bicolor* (Sb), *Setaria italica* (Si), *Setaria viridis* (Sv), *Saccharum spontaneum* (Ss), and *Zea mays* (Zm). The tree is rooted with *Oryza sativa* Rubisco large subunit (OsRbcL). Branch lengths represent substitutions per site of the alignment sequence.

**
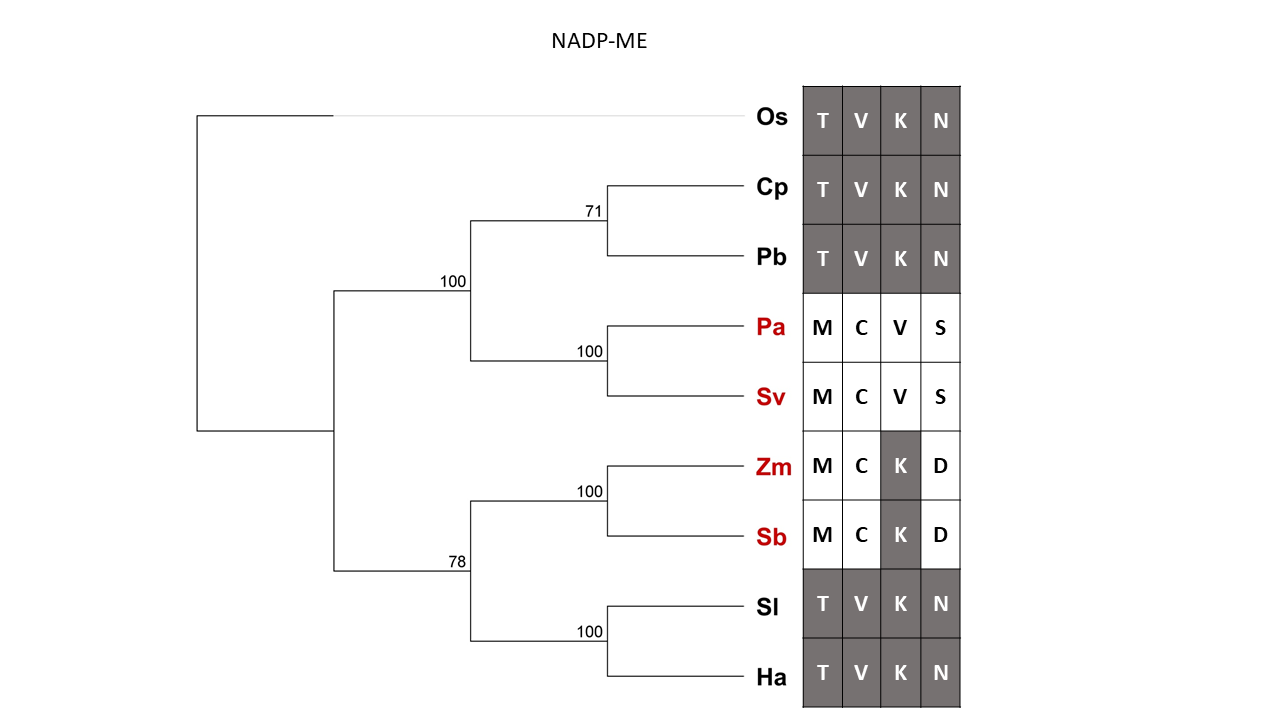
Figure S7 C_4_-dependent evolution of NADP-ME.**

NADP-ME protein sequences were examined four C_4_ species, *Panicum antidotale* (Pa), *Sorghum bicolor* (Sb), *Setaria viridis* (Sv) and *Zea mays* (Zm) and five C_3_ species, *Hymenachne amplexicaulis* (Ha), *Panicum bisulcatum* (Pb), *Steinchisma laxum* (Sl) and *Cyrtococcum patens* (Cp). Codons represent aa regions which show levels of trait-dependent evolution. The different colours indicate changes in all (white) or some (light grey) C_4_ species. The tree was constructed using boot strap BLOSSOM96, 1.1 million repetitions. The tree was rooted to Oryza sativa (Os).

**Figure S8 Leaf to seed expression ratio of sugar sensor genes in C­_4_ and C_3_ grasses.**

RMA or TPM values from either microarray or RNAseq data of sugar sensor genes were used to calculate leaf to seed ratios from the C_4_ grasses *Zea mays* and *Panicum millaceum* and the C_3_ grasses *Oryza sativa* and *Brachypodium distachyon* (Yue *et al.*, 2016; Sekhon *et al.*, 2011; Jain *et al.*, 2007; Sibout *et al.*, 2017). Each species consisted of three biological replicates for each tissue sampled. Data represents the mean leaf to seed ratios of genes from C_4_ and C_3_ grasses (n=3). Error bars represent the SEM. Ratios <1 indicate expression of the gene predominating in the seed whereas >1 indicate expression predominating in the leaf. Broken line indicates 1. Red asterisks represent significant difference to 1 and predominating in the leaf. Blue asterisks represent significant difference to 1 and predominating in the seed. Related to Figure 1.

**Table S2 Leaf to seed expression ratio of sugar sensor genes in C_4_ and C_3_ grasses. Paired** Student’s t-test compared to 1 p-values. Related to Figure 1 and Figure S8.

| **Gene** | ***Zea mays*** | ***Panicum miliaceum*** | ***Oryza sativa*** | ***Brachypodium distachyon*** | **C_4_ grasses** | **C_3_ grasses** |
| --- | --- | --- | --- | --- | --- | --- |
| ***TOR*** |  |  | 0.158 | 0.158 |  | 0.237 |
| ***LST8-1*** | 0.017 | 0.040 | 0.083 | 0.083 | 0.067 | 0.076 |
| ***RAPTOR1*** | 0.036 | 0.331 | 0.128 | 0.128 | 0.094 | 0.063 |
| ***RAPTOR2*** | 0.037 | 0.894 | 0.069 | 0.069 | 0.708 | 0.069 |
| ***RAPTOR3*** | 0.004 | 0.688 |  |  | 0.169 |  |
| ***SnRK1α1*** | 0.073 | 0.255 | 0.260 | 0.260 | 0.208 | 0.342 |
| ***SnRK1α2*** | 0.006 | 0.023 | 0.450 | 0.450 | 0.163 | 0.830 |
| ***SnRK1α3*** | 0.602 | 0.836 | 0.450 | 0.450 | 0.838 | 0.719 |
| ***SnRK1β1*** | 0.022 | 0.940 | 0.109 | 0.109 | 0.495 | 0.282 |
| ***SnRK1β2*** | 0.179 | 0.916 | 0.106 | 0.106 | 0.800 | 0.173 |
| ***SnRK1β3*** | 0.013 | 0.017 | 0.138 | 0.138 | 0.051 | 0.138 |
| ***SnRK1γ1*** | 0.123 | 0.167 | 0.092 | 0.092 | 0.160 | 0.166 |
| ***SnRK1γ2*** | 0.560 | 0.017 | 0.957 | 0.957 | 0.109 | 0.905 |
| ***SnRK1βγ1*** | 0.000 | 0.240 | 0.118 | 0.118 | 0.254 | 0.004 |
| ***HXK5*** | 0.066 | 0.403 | 0.060 | 0.060 | 0.267 | 0.060 |
| ***HXK6*** |  | 0.864 | 0.085 | 0.085 | 0.864 | 0.085 |
| ***TPS1*** | 0.007 | 0.188 | 0.139 | 0.139 | 0.170 | 0.289 |
| ***TPP1*** | 0.020 | 0.266 | 0.017 | 0.017 | 0.230 | 0.037 |
| ***TRE*** | 0.052 | 0.160 | 0.233 | 0.233 | 0.151 | 0.140 |

**Table S3 Bundle sheath and mesophyll cell sugar sensor gene expression in C_4_ grasses.** Paired Student’s t-test p-values. Related to Figure 6.

| **Gene** | ***Zea mays*** | ***Sorghum bicolor*** | ***Setaria viridis*** | ***Setaria italica*** | ***Panicum hallii*** | **C_4_ grasses** |
| --- | --- | --- | --- | --- | --- | --- |
| ***TOR*** | 0.074 | 0.043 | 0.378 | 0.174 | 0.001 | 0.130 |
| ***LST8-1*** | 0.345 | 0.064 | 0.002 | 0.001 | 0.076 | 0.703 |
| ***LST8-2*** |  |  | 0.004 | 0.680 |  |  |
| ***RAPTOR1*** | 0.361 | 0.062 | 0.961 | 0.050 | 0.004 | 0.084 |
| ***RAPTOR2*** | 0.466 | 0.045 | 0.754 | 0.989 | 0.010 | 0.202 |
| ***RAPTOR3*** | 0.066 | 0.031 |  | 0.989 |  |  |
| ***SnRK1α1*** | 0.561 | 0.005 | 0.093 | 0.005 | 0.000 | 0.462 |
| ***SnRK1α2*** | 0.210 | 0.015 | 0.072 | 0.012 | 0.000 | 0.112 |
| ***SnRK1α3*** | 0.014 | 0.459 | 0.038 | 0.008 | 0.001 | 0.099 |
| ***SnRK1β1*** | 0.087 | 0.041 | 0.286 | 0.328 | 0.153 | 0.049 |
| ***SnRK1β2*** | 0.357 | 0.206 | 0.030 | 0.032 | 0.354 | 0.891 |
| ***SnRK1β3*** | 0.568 | 0.015 | 0.306 | 0.052 | 0.015 | 0.697 |
| ***SnRK1γ1*** | 0.668 | 0.190 | 0.360 | 0.001 | 0.177 | 0.338 |
| ***SnRK1γ2*** | 0.123 | 0.041 | 0.691 |  | 0.401 |  |
| ***SnRK1βγ1*** | 0.947 | 0.041 | 0.090 | 0.016 | 0.034 | 0.619 |
| ***SnRK1βγ2*** | 0.871 |  |  |  |  |  |
| ***HXK5*** | 0.141 | 0.010 | 0.205 | 0.009 | 0.004 | 0.206 |
| ***HXK6*** | 0.389 | 0.023 | 0.094 | 0.157 | 0.016 | 0.192 |
| ***TPS1*** | 0.214 | 0.005 | 0.012 | 0.002 | 0.001 | 0.006 |
| ***TPP1*** | 0.005 | 0.029 | 0.048 | 0.692 | 0.014 | 0.787 |
| ***TRE*** | 0.636 | 0.171 | 0.809 | 0.058 | 0.034 | 0.667 |
